# PGM1 deficiency is linked to sarcomeric and mitochondrial dysfunction in patient-derived iPSC-cardiomyocytes

**DOI:** 10.1186/s12967-026-07808-9

**Published:** 2026-02-21

**Authors:** Silvia Radenkovic, Graeme Preston, Rohit Budhraja, Irena Muffels, Anna Ligezka, Nathan P. Staff, Ron Hrstka, Bijina Balakrishnan, Rameen Shah, Sanne Verberkmoes, Ibrahim Shammas, Inez Bosnyak, Kyle M. Stiers, Kent Lai, Lesa J. Beamer, Akhilesh Pandey, Eva Morava, Tamas Kozicz

**Affiliations:** 1 Department of Clinical Genomics, Mayo Clinic, Rochester, MN 55905 USA; 2 Renaissance School of Medicine, Stony Brook University, Stony Brook, NY 11794 USA; 3 Department of Genetics, Section Metabolic Diagnostics, University Medical Center Utrecht, Utrecht, 3584 EA The Netherlands; 4 Department of Genetics and Genomics Sciences, Icahn School of Medicine at Mount Sinai, New York City, NY 10029 USA; 5 Department of Laboratory Medicine and Pathology, Mayo Clinic, Rochester, MN 55905 USA; 6 Department of Neurology, Mayo Clinic, Rochester, MN 55905 USA; 7 Division of Medical Genetics, Department of Pediatrics, University of Utah, Salt Lake City, UT 84108 USA; 8 Manipal Academy of Higher Education (MAHE), Manipal, Karnataka 576104 India; 9 Department of Biophysics, University of Pecs Medical School, Pecs, 7624 Hungary; 10 Biochemistry Department, University of Missouri, Columbia, MO 65211 USA; 11 Department of Anatomy, University of Pecs Medical School, Pecs, 7624 Hungary

**Keywords:** Phosphoglucomutase-1, Cardiac dysfunction, Z-disk, Mitochondrial dysfunction, PGM1-CDG

## Abstract

**Background:**

PGM1-congenital disorder of glycosylation (PGM1-CDG) is frequently associated with cardiomyopathy. Although galactose therapy corrects glycosylation defects, cardiac dysfunction typically persists, suggesting a glycosylation-independent mechanism. Recent evidence of mitochondrial abnormalities in PGM1-deficient human and murine heart, together with the association of PGM1 with the Z-disk protein LDB3 (ZASP/Cypher), suggests a critical role for PGM1 in cardiomyocyte structural and energetic homeostasis. We hypothesized that PGM1-related cardiomyopathy arises from a glycosylation-independent disruption of Z-disk–mitochondrial coupling driven by loss of PGM1–LDB3 interactions, resulting in mitochondrial energy failure and impaired contractile function.

**Methods:**

Induced pluripotent stem cell–derived cardiomyocytes (iCMs) were generated from PGM1-deficient patient fibroblasts. Multielectrode array (MEA) recordings, untargeted (glyco)proteomics, and pathway analysis were performed to assess functional and molecular changes. Key findings were validated using tracer metabolomics and mitochondrial respiration assays.

**Results:**

PGM1-deficient iCMs exhibited reduced beating frequency, impaired contractility, and prolonged contraction kinetics. Proteomic analyses revealed depletion of Z-disk components, including LDB3. AlphaFold3 structural modeling predicted a direct interaction between PGM1 and LDB3, implicating PGM1 in Z-disk integrity, which was confirmed in vitro. In addition, mitochondrial proteins were severely depleted, prompting us to investigate mitochondrial function. Functional validation confirmed extensive metabolic rewiring, energy depletion, and severely impaired mitochondrial respiration. Finally, the in silico drug repurposing identified possible therapeutic options that could target PGM1-deficient cardiomyopathy.

**Conclusion:**

Our data suggests PGM1 is key regulator of cardiomyocyte function, linking sarcomeric Z-disk integrity with mitochondrial metabolism. These mechanistic insights offer a foundation for developing targeted therapies for PGM1-CDG and potentially other cardiomyopathies involving Z-disk dysfunction.

**Graphical Abstract:**

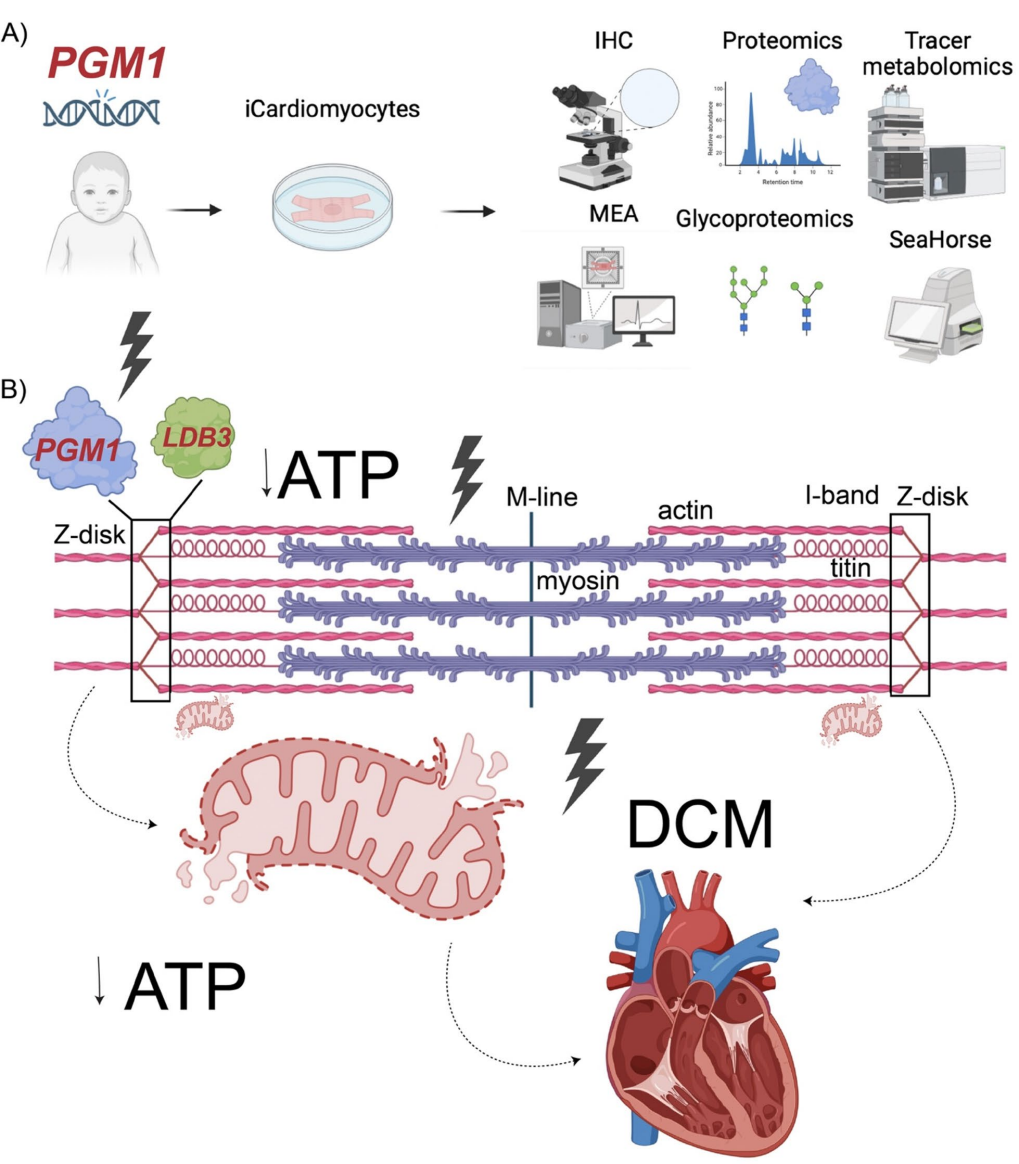

**Supplementary Information:**

The online version contains supplementary material available at 10.1186/s12967-026-07808-9.

## Background

**Fig. 1:**
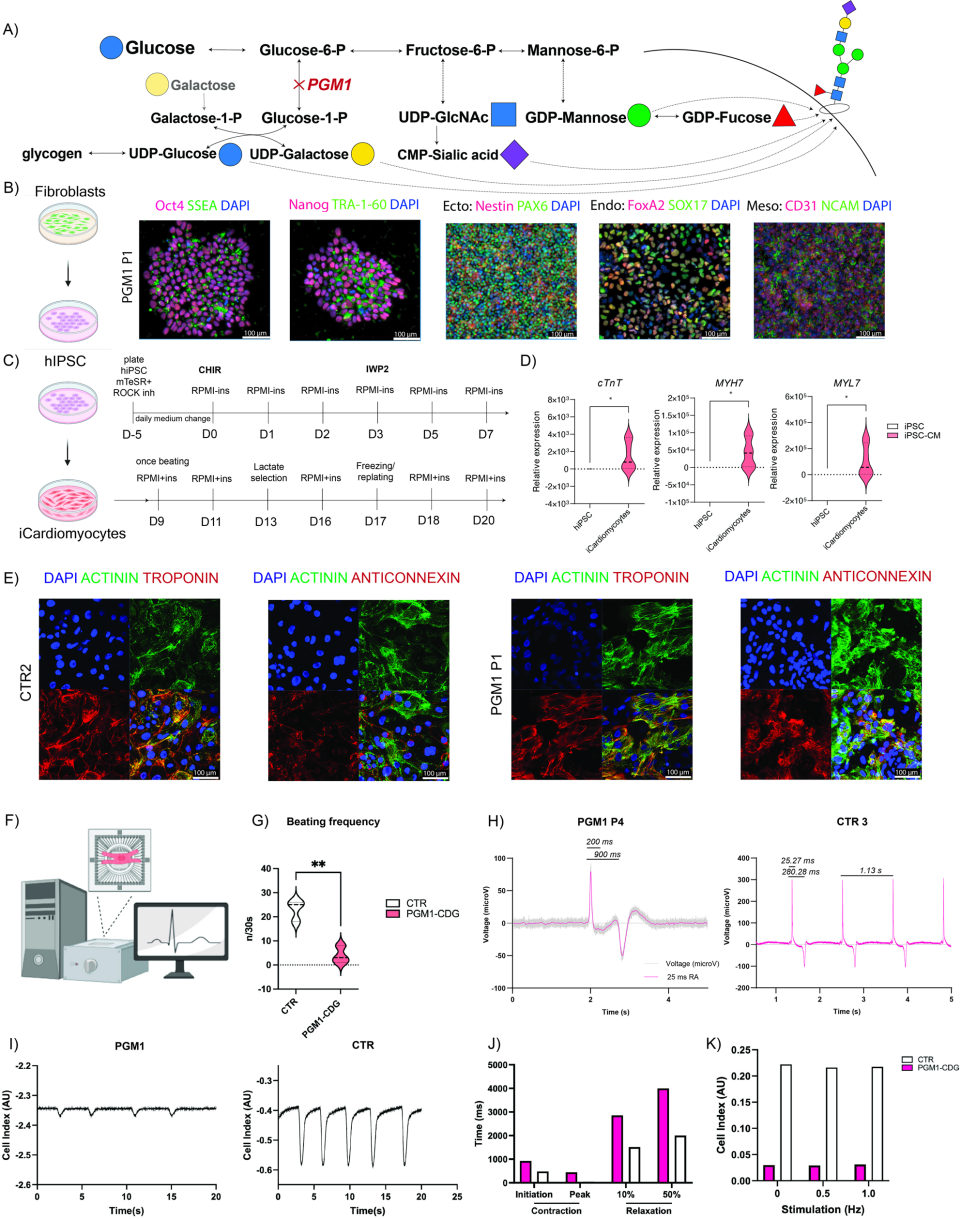
**A**) Schematic representation of **B**) The successful reprogramming of fibroblasts into human induced pluripotent stem cells (hiPSC) was validated by pluripotency markers (Oct4, SSEA, Nanog, TRA-1-60) and the three-germ line differentiation: ectoderm (ecto- Nestin, PAX6), endoderm (endo- FoxA2, SOX17), and mesoderm (meso- CD31, NCAM). **C**) hiPSC were differentiated into iCM following the chemically defined protocol. Scale bar shows 100 µm. Cardiomyocyte markers were assessed in control and PGM1 iCM by **D**) RT-qPCR for cardiac troponin (cTnT), myosin heavy chain 7 (MYH7) and myosin light chain 7 (MYL7); and **E**) Immunohistochemistry (IHC) for actinin (green), cardiac troponin (red), and anti-connexin43 (anticonnexin; red). Scale bar shows 100 µm. **F**) Schematic representation multielectrode array (MEA) of iCM; **G**) PGM1-deficient iCM show decreased beating rate compared to healthy CTR. PGM1- iCM *n* = 3, CTR iCM *n* = 3. **H**) Representative MEA readouts for healthy PGM1 deficiency is linked to sarcomeric and mitochondrial dysfunction in patient-derived iPSC-cardiomyocytes CTR (top) and PGM1-deficient iCM. X- axis shows the time of recording in seconds (s). Y-axis shows the voltage (µV). **I**) PGM1 iCMs displayed a profound reduction in contractility relative to the control **J**) Stimulation with 0.3 ms pulses of 1200 mV at either 0.5 or 1.0 Hz in PGM1 and CTR iCM did not show any improvement in contraction amplitude. **K**). PGM1 iCMs displayed increased time between application of the stimulation and both contraction initiation and peak contraction. PGM1-iCMs also displayed increased time between peak contraction and both 10% and 50% relaxation. SD is shown; p value marked with * corresponds to *p* < 0.05; ** corresponds to p < 0.001

Phosphoglucomutase-1 congenital disorder of glycosylation (PGM1-CDG) (OMIM: 614921) is a multisystem disease caused by biallelic pathogenic *PGM1* variants, affecting glycogen metabolism, glycolysis, and glycosylation (Fig. 1A) [1, 2]. While clinical presentation often includes liver involvement, dysmorphic features, and coagulation abnormalities, cardiac dysfunction is the most severe, often lethal presentation in PGM1-CDG [1–4].

The age of onset of cardiac complications in PGM1-CDG is variable and can occur within the first 5 years [1–8]. The most frequent cardiac complication in PGM1-CDG is dilated cardiomyopathy, but enlarged left ventricle, left-ventricular non-compaction cardiomyopathy, hypertrophy, and ECG abnormalities including tachycardia and prolonged QT have also been described [3–5, 7, 9–11]. Cardiac failure resulting in cardiac arrest and early death was reported in more than 12% of previously reported cases [9].

Despite effective correction of glycosylation defects and improvement of systemic clinical features with oral galactose supplementation in PGM1-CDG [1, 3, 5, 10, 12, 13], cardiac manifestations remain largely refractory to treatment [3, 5, 10, 11, 13]. This disconnect highlights a critical unmet research need to determine whether PGM1 has a direct, non-glycosylation role in maintaining cardiomyocyte contractile function and whether PGM1 deficiency itself is a primary driver of cardiomyopathy.

Cardiac presentation is uncommon in other CDG [4, 14], on the other hand, it has been reported in almost 50% of PGM1-CDG individuals. The genotype-phenotype correlation in PGM1 deficiency was partially elucidated by the identification of two PGM1 isoforms: PGM1-1 and PGM1-2 [1–4, 15]. PGM1-1 is ubiquitously expressed, while PGM1-2 is specifically expressed in muscle and heart tissue [9]. The patients whose pathogenic variants affect PGM1-2 isoform have the highest occurrence of muscle and cardiac presentation [9], suggesting PGM1-2 plays a crucial role in the heart. PGM1-2 also features a unique binding motif, in comparison to PGM1-1, suggesting a potential cardiac-specific binding partner [9]. Analogously, PGM1 has been linked to LDB3 [16], a key component of the sarcomeric Z-disk [16, 17]. PGM1 was first identified as a potential biding partner of LDB3 in a yeast screen [16]. In addition, immunohistochemistry in rat cardiomyocytes showed PGM1 colocalizes with LDB3 in the sarcomere, while mutant LDB3 showed reduced binding to PGM1 resulting in sarcomere disruption [16]. Since, pathogenic *LDB3* variants lead to sarcomere disruption and dilated cardiomyopathy [16, 17], the role of PGM1 in LDB3 cardiomyopathy was suggested [16]. Despite previous studies linking PGM1 and LDB3, it remains unclear whether PGM1 is a required component of the LDB3 sarcomeric network in human cardiomyocytes, and whether its deficiency alone is sufficient to drive cardiomyopathic phenotypes.

A potential role for mitochondrial dysfunction in PGM1-associated cardiomyopathy has been suggested by the observation of abnormal mitochondrial morphology in the explanted heart of a one-year-old PGM1-CDG patient with severe cardiomyopathy who underwent heart transplantation [18]. To further investigate PGM1-associated cardiomyopathy, a cardiac-specific PGM1 knockout mouse model was developed [18]. This model recapitulated some of the structural and functional mitochondrial abnormalities observed in patient [18], however, these experiments were performed in PGM1 KO mouse models, which do not fully recapitulate physiology as well as genetic diversity of PGM1-CDG.

Therefore, the precise mechanisms by which PGM1 deficiency impairs cardiac and mitochondrial function and whether these effects reflect direct cardiomyocyte-specific roles of PGM1 remain unresolved.

We hypothesized that PGM1, independent of its canonical role in glycosylation, has a cardiomyocyte-specific function at the sarcomeric Z-disk through its interaction with LDB3, and that loss of PGM1 impairs contractile function, and secondarily compromises mitochondrial bioenergetics, thereby driving cardiomyopathy. Using multi-omics and functional analyses in patient-derived induced pluripotent stem cell-cardiomyocytes (iCMs), we show that PGM1 deficiency leads to impaired contractility, altered excitation–contraction coupling, depletion of sarcomeric and mitochondrial proteins (including the Z-disk protein LDB3), metabolic alterations and mitochondrial bioenergetic failure. Structural modeling and in vitro binding assays support a direct PGM1-LDB3 interaction, highlighting the dual role of PGM1 in sarcomeric integrity and metabolic function. Together, these findings suggest a previously unrecognized mechanism underlying PGM1-associated cardiomyopathy and provide a further framework for developing candidate therapeutic strategies.

## Methods

### Cell culture of patient-derived fibroblasts

Fibroblasts from four patients were collected as part of clinical care via skin-punch biopsy and residual samples were stored in the Mayo Clinic FCDGC biobank (Mayo IRB: *16-004682*). Informed consent was obtained and recorded. Fibroblasts were maintained in MEM medium (Gibco, 11095080) supplemented with 10% FBS (Cardinal Healthcare M7201-127) and 1% Anti-Anti (Gibco, 15240062) in the incubator at 37 °C, 5% CO_2_. Routine mycoplasma testing was performed.

### Generation and maintenance of human induced pluripotent stem cells (hiPSC)

hiPSCs were generated from patient fibroblasts following standardized, previously reported methods [19]. Briefly, Sendai virus Cytotune 2.0 kit (ThermoFisher) was used to generate PGM1-CDG (P1, P2, P3, P4) and healthy control hiPSC (GM8399, GM1651 Coriell Institute for Medical Research, NJ, USA). Absence of chromosomal abnormalities was confirmed by karyotype G-banding. Markers of pluripotency were assessed by flow cytometry, immunohistochemistry (Oct 4, SSEA, Nanog, Tra-1-60), and three-germ layer (trilineage) differentiation. Mycoplasma testing was routinely performed. Only the hiPSC clones meeting all the criteria were selected. All hiPSCs were maintained in mTesr Plus medium supplemented with 10% mTeSR supplement and 1% Anti-Anti (Gibco) on 60 mm dishes coated with 1 mg/ml Geltrex matrix (ThermoFisher, A1413302) in the incubator at 37 °C, 5% C_2_.

### Differentiation of hiPSC into iCMs

Differentiation of hiPSC into the hiPSC-derived cardiomyocytes (iCMs) was performed based on the previously described chemically defined cardiomyocyte differentiation protocol [20, 21]. The protocol was optimized for each hiPSC cell line, to ensure the optimal generation of cardiomyocytes. The outline of the protocol is shown in Fig. 1. Briefly, hiPSC were maintained in 60 mm dishes in 10% mTeSR supplement (Stem Cell Technologies) and 1% Anti-Anti (Gibco). Once confluent, hiPSC were seeded into 6-well plates coated with 1 mg/ml Geltrex (ThermoFisher, A1413302) in mTeSR plus medium supplemented with 10µM ROCK inhibitor (Y-27632, Torcis 5148). Once the cells reached 90–95% confluence, they were treated with CHIR99021 (Tocris, 4423) in RPMI 1640 (Gibco, 11875093) supplemented with B27-insulin (Thermo Fisher Scientific, A1895601) (referred to as RPMI- insulin) for three days (Day0-Day2). The concentration was optimized for each cell line and between 3 and 8 µM was used. Next, the medium with CHIR was removed, the cells were washed with DPBS (Gibco) and the cells were treated with 5µM IWP2 in RPMI-insulin for 48 h (day 3–5). After IWP2 treatment, the medium was removed, the cells washed with DPBS and new RPMI-insulin medium added every 48 h, until beating is observed (usually between D7-D14). The cells that did not start beating after two weeks in culture were discarded. Once the beating was observed, RPMI 1640 medium supplemented with B27 with insulin (ThermoFisher, 17504044) (from here referred to as RPMI+insulin) was added to the cells. To purify the cardiomyocyte culture and remove other cell types, lactate selection was performed by incubating cells with RPMI 1640 without glucose (Gibco, 11879020), supplemented with Sodium DL-Lactate Solution (Sigma, L4263), Recombinant Human Albumin (Sigma, A9731) and L-ascorbic Acid (Sigma, A8960) until majority of the cells that survived are beating (1–3 days). The medium was then switched back to RPMI+ insulin for maintenance. Finally, the cells were dissociated from the plates with TrypLE (Thermo Fisher Scientific, A1217703), collected in RPMI+insulin supplemented with 10% Knock-Out Serum (KOSR, ThermoFisher 10829018) and 10 µM ROCK inhibitor Y-27,632. The cells were counted and pelleted at 800 rpm, 6 min, room temperature (RT) before freezing them in 90%, 10% DMSO (Sigma, D2650), 10 µM ROCK inhibitor. Approximately 300microL of freezing medium was used for each million of cells frozen. For all the subsequent experiments, the cells were thawed in RPMI+ insulin supplemented with 10% Knock-Out Serum (KOSR, ThermoFisher 10829018) and 10 µM ROCK inhibitor Y-27,632 (1:10), counted and seeded for specific experiments (see below). The viability after thawing ranged from 70 to 90%. Immunohistochemistry, quantitative PCR analysis, multielectrode array (MEA) and contractility studies were used to further phenotype PGM1-deficient and control iCardiomyocytes (iCMs) based on previously published protocols (see supplementary materials for details).

### Untargeted proteomics and N-glycoproteomics

Untargeted proteomics and N-glycoproteomics were performed to assess the (glyco)protein expression changes between PGM1-deficient and CTR iCMs based on previously published protocols [18, 19, 22] (see supplementary materials for details).

### Untargeted proteomics and glycoproteomics data analysis

Proteomics and N-glycoproteomics data analysis was performed as described previously [18, 19, 22]. The proteomics data were searched using Sequest search engine in Proteome Discoverer 3.0 against the human Uniprot protein database. The N-glycoproteomics data using the publicly available software pGlyco version 3 with an in-built N-glycan database for identifying glycans and human Uniprot protein database for identifying peptide sequence. Two missed cleavages were allowed for both proteomics and glycoproteomics analysis. Error tolerance for precursor and fragment ions were set to 10 ppm and 0.02 Da, respectively, for proteomics and 10 ppm and 20 ppm, respectively, for glycoproteomics. Cysteine carbamidomethylation was set as fixed modification, whereas oxidation of methionine as variable modification. False discovery rate (FDR) was set to 1% at the peptide-spectrum matches (PSMs), peptide, protein and glycopeptides levels. For proteomics, quantitation of peptides across PGM1-CDG and control fibroblasts was done using TMT reporter ion intensities using “reporter ion quantifier” node. To quantify glycopeptides, reporter ion quantification was performed for glycoproteomics raw files in Proteome Discoverer and glycopeptide IDs obtained from pGlyco were matched with quantitation on a scan-to-scan basis (MS/MS). For mitochondrial proteins, annotated gene list was generated from MitoCarta 3.0 [23].

### MitoCarta analysis

To assess for a depletion of the MitoCarta [23] protein pool in our proteomics dataset, the number of MitoCarta protein species detected with a FC greater or less than 1 relative to controls was counted and compared to the number of total protein species detected displaying a FC greater than or less than 1 relative to controls. These counted proteins were then compared using Fisher’s exact test. This protocol was repeated for the detected subunits of mitochondrial electron transport chain complex I, complex II, complex III, complex IV, and complex V, and the mitochondrial ribosome, as well as all proteins involved with mitochondrial membrane integrity with an associated mitochondrial disease. These subunit proteins abundances and counts were compared to both the MitoCarta and total protein pools. Additionally the average FC relative to controls of all MitoCarta proteins, the detected subunits of mitochondrial electron transport chain complex I, complex II, complex III, complex IV, and complex V, and the mitochondrial ribosome, as well as all proteins involved with mitochondrial membrane integrity with an associated mitochondrial disease [24] was compared to the average FC of all protein species detected relative to controls using Kruskal-Wallis test.

### Ingenuity Pathway Analysis (IPA)

The functional analyses of the proteomics results were generated through the use of IPA [25] (QIAGEN Inc., https://www.qiagenbio-informatics.com/products/ingenuity-pathway-analysis). The IPA analysis was conducted using IPA default settings was performed on the top significantly changing proteins identified by proteomics. Log2 fold change of proteins with p-value of < 0.05 was used to calculate IPA analysis-ready molecules, and z-scores, representing the activation state of each canonical and disease pathway (z-score ≥ 2 significantly activated and z-score ≤ -2 significantly inhibited). All proteins with significant p-value < 0.05 were analyzed. IPA analysis was limited to databases related to humans. To avoid bias, no tissue specificity was selected in the analysis. In addition, toxic (tox) functions analysis was performed by IPA, which catalogs the genes/molecules/proteins known to be involved with specific type of toxicity and their causal associations, when known. These analyses include associations to organ injuries, clinical chemistry and hematology assays, and pathway-related endpoints such as mitochondrial function etc. The molecules and their associations with different toxicity endpoints and pathologies are hand-curated by IPA, and include renal, hepatic and cardiac injury, etc.

### GSEA analysis

The GSEA analyses of the proteomics results were generated using R (Version 4.3) an R-studio (V2023.09.1 + 494). For GSEA, a pre-ranked list based on log2foldchange*-log10(p-value) was used as input for the gseGO() function. Biological Process of GO Ontology was used as pathways. Benjamin Hochberg was used to calculate statistics. The adjusted p-value cutoff was set at 0.001 for upregulated pathways and 0.0001 for downregulated proteins. Semantically similar pathways were manually curated. A complete list of pathways can be found in Additional Table X. The following packages were used to create the graphs: ggplot2 (3.5.1), ClusterProfiler (4.10.1) [26].

### Targeted tracer metabolomics experiments

Targeted tracer metabolomics was performed to assess the differences in the central carbon metabolism of PGM1-deficient and CTR iCMs based on previously described protocols [5, 27, 28] (see supplementary materials for details) [19, 28–30]. Metabolite abundances were normalized to protein concentration and internal standards. Absolute quantification was not performed. Relative values were established using healthy control iCMs as reference.

### Fractional contribution of ^13^C_6_-glucose and isotopologues statistical analysis

Fractional contribution of ^13^C_6_-glucose is defined as a percentage of ^13^C_6_-glucose contributing to the pool of specific measured metabolite. Isotopologue labeling (positional labeliing) of ^13^C_6_-glucose is defined as a percentage of numbers of carbons labeled by ^13^C_6_-glucose in a specific metabolite (m0-mn, where n signifies the number of carbons in a specific metabolite). Visualization of the fractional contribution (FC) of ^13^C_6_-glucose in PGM1-CDG and control iCMs, was performed using TraVis Pies [29]. TWO-way ANOVA was used to assess the significant differences between PGM1-deficient and CTR iCMs in overall isotopologue labeling and significant difference in the isotopologues distribution based on the genotype (interaction between genotype and isotologue distribution). Multiple comparisons with Sidak correction analysis was used to assess significant differences between PGM1 deficient and CTR iCMs in specific isotopologues. The results of the statistical analysis are provided in additional data.

### Metabolic pathway analysis

MetaboAnalyst 5.0 [30] was used to perform pathway analysis on metabolomics data to analyze and visualize the pathway trends. Metabolomics data containing Fold Change information and metabolite IDs was analyzed by standard MetaboAnalyst parameters. These included: enrichment method- global test; topology measure relative-betweenness centrality, and the metabolites were compared to all the compounds in the KEGG pathway library (Homo Sapiens). False Rate Discovery (FDR < 0.05) was applied. Pathway relevance was scored based on the p-value and pathway impact.

### Mitochondrial respiration studies

Seahorse XFe96 Extracellular Flux Analyzer (Agilent, Santa Clara, CA, USA) and XF Cell Mito Stress Test kit (Agilent, 103015-100) were used to investigate the mitochondrial respiration of PGM1-deficient and CTR iCMs, as previously described in patient fibroblasts [31]. The seeding density and inhibitor concentrations were specifically optimized for iCMs. iCMs were first generated and frozen based on the above-mentioned protocols. For the Seahorse the experiments both CTR and PGM1 iCMs were simultaneously thawed as described above. The cells were counted with the automated Cell Counter (Countess 3, Invitrogen) using trypan blue cell viability dye (Invitrogen) to ensure that the same number of viable cells were seeded. The cells were seeded at 40,000 (viable) cells per well in RPMI+ insulin supplemented with 10% KOSR and 10µM ROCK inhibitor in a 96-well Seahorse microplate (day 0). Each cell line was seeded in 8-well replicates. The viability of the cells and the beating was assessed daily. The medium was refreshed the following day (day 1), and once again after 48 h (day 3). Then, 48 h before the measurements, the medium was changed to RPMI+ insulin containing 5.5 mM (physiologic concentration) glucose (day 5). On the day of the measurement (day 7), the cells were washed with DPBS and the medium was replaced with XF Base Medium Minimal RPMI (Agilent, 103576-100) supplemented with 10 mM XF seahorse glucose (Agilent, 103577-100), 1 mM XF seahorse pyruvate (Agilent, 103578-100), and 2 mM L-glutamine according to the manufacturer’s instructions. After the initial basal respiration measurements, the specific inhibitors used to assess mitochondrial respiration were added in following order: port A 2.5 µM oligomycin, port B 2.0 µM carbonyl cyanide phenylhydrazone (FCCP), port C 0.5 µM rotenone + antimycin A (Agilent, 103015-100). After the measurements, viability and the cell number were assessed again by using the cell membrane-permeable nuclear staining compound (Hoechst 33342) and performing cell counting using Celigo imaging cytometer (Beckman). The experiment was repeated 3 times using new cells each time, avoiding freeze-thaw cycles. The results were normalized to the cell number and citrate synthase (CS) activity, a proxy readout for mitochondrial mass [32], described below. Basal respiration, Oxygen consumption rate (OCR), proton efflux rate (PER), extracellular acidification rate (ECAR), maximal respiration rate and ATP-linked respiration were analysed based on the manufacturer’s protocol and previously described methods [31, 33].

### Citrate synthase (CS) activity/abundance

CS is a matrix enzyme of the TCA/Krebs cycle that catalyses the conversion of oxaloacetate and acetyl-CoA to citrate and CoA. CS is highly enriched in the mitochondrial matrix and is thus commonly used as a proxy readout for mitochondrial mass, which might be a confounding factor in assessing the mitochondrial respiration [32]. CS measurements were performed as previously described [31, 33]. Briefly, cell lysates were incubated with acetyl-CoA and oxaloacetate in the presence of DTNB (5’,5’dithiobis-(2nitrobenzoaat)). Precipitation of acetyl-CoA and oxaloacetic acid to citrate releases S-CoA, which cleaves DTNB (colorless) to TNB^2-^ (yellow). Increase in absorption at 412 nm was assessed as CS activity. For a blank measurement, the reaction was performed in the absence of oxaloacetate.

### In silico prediction of LDB3- PGM1 interaction using AlphaFold3

**Fig. 2:**
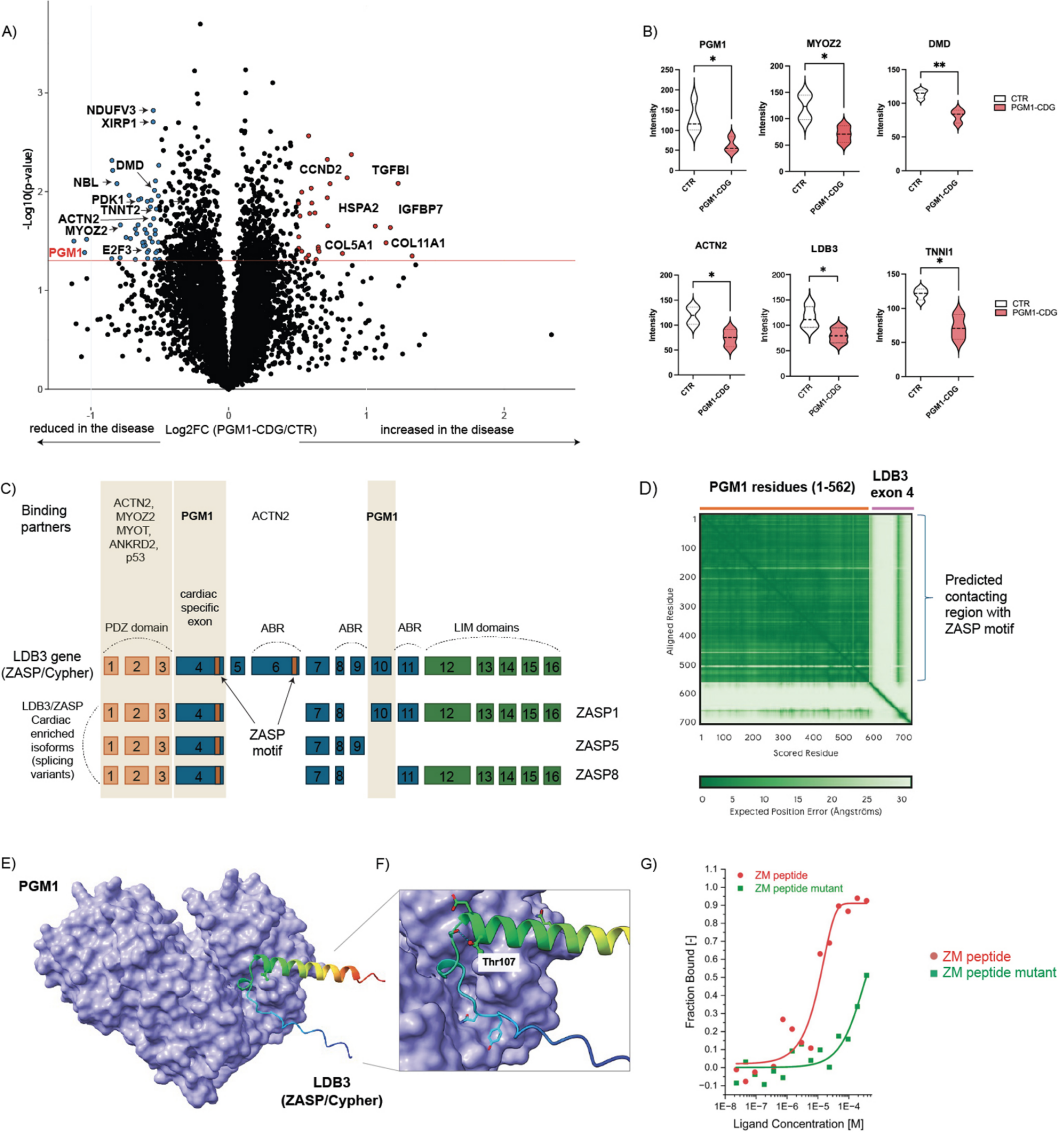
Sarcomeric Protein Dysregulation and Potential Z-disk Impairment in PGM1-Deficient iCMs. **A**) Volcano plot showing differentially abundant proteins in PGM1-deficient iCM compared to healthy control iCM (CTR). X-axis shows log_2_ fold change (FC) (PGM1-deficient/CTR) and Y-axis represents the -log10 of p-value. The horizontal red line represents the cutoff for significance [-log10(0.05)]. The most significantly changing proteins are marked in either red (> 1.3 FC, *p* < 0.05) or blue (< 0.7, *p* < 0.05). **B**) Violin plots showing some of the top significantly changing proteins involved in cardiomyocyte machinery in PGM1-deficient iCM compared to CTR. Y-axis represents the ion intensity of TMT channels. PGM1 *n* = 3, t = 1; CTR *n* = 4, t = 1. Violin plots are shown with standard deviation (SD). Detailed statistical analysis can be found in the supplemental information. **C**) Schematic of the LDB3 gene (16 exons) that encodes various isoforms of ZASP/Cypher. The three splicing variants of ZASP (isoforms) are also shown with the cardiac specific exon 4 and ZASP motifs on exon 4 and 6 indicated by orange box. Regions with known interactions with PGM1 (exons 4 and 10), as well as ACTN2, MYOZ2, MYOT, ANKRD2 and p53 are highlighted. ABR- actin binding region. **D**) The predicted alignment error (PAE) matrix from AF3 for the top scoring model of the PGM1 and Exon 4 complex, which had ipTM and pPTM values of 0.45 and 0.80, respectively. **E**) A figure showing the predicted interaction between PGM1 and the ZASP motif of Exon 4 of LBD3 (Zasp/Cypher). PGM1 is shown in a surface representation (purple); residues 85–128 of Exon 4 are shown as a ribbon, in a color ramp from blue (N-terminus) to red (C-terminus). The ZASP motif is found within the helical region (green) and Thr107 is shown in a space filling model. A Thr to Ile mutant of residue 107 has been reported to impair interactions with PGM1 [16]. **F**) A close-up view of the potential PGM1 interaction with the ZASP motif of Exon 4. The location of Thr107 near the N-terminus of the helix is highlighted, along with a hydrogen bond between the Thr107 side chain and a preceding residue. This interaction suggests that Thr107 may play a role in stabilizing the helical conformation of the ZASP motif and that mutation to Ile could disrupt the helical structure and impair binding to PGM1. **G**) High affinity in vitro interactions between exon 4 of LBD3 (ZASP/Cypher) and PGM1

AlphaFold3 (AF3) [34] was used to predict potential interactions between PGM1 and Zasp/Cypher (LBD3). We focused on potential interactions with Exon 4 of Zasp/Cypher, which have been previously identified [16]. Exon 4 contains the conserved ZASP motif (residues 189–214 of LBD3 isoform 1), implicated in protein interactions. AF3 used on PGM1 predicts structure with high confidence, as expected give that its crystal structure is known. AF3 used on Exon4 (of unknown structure) indicates mostly disordered regions but does predict a structured region with high confidence that corresponds approximately to the ZASP motif. In predictions of a potential PGM1-Exon 4 complex, the top scoring model returned values of 0.45 for ipTM (just below the 0.5 level suggesting a correct prediction) and pTM of 0.80 (matching the 0.8 cutoff for a confident high-quality prediction) using the PGM1 sequence (isoform 1). Moreover, the predicted aligned error (PAE) for the interaction between PGM1 and the ZASP motif of Exon 4 is low (< 5 Å) supporting an accurate prediction (Fig. 2D). (As noted in AF3 documentation, PAE for may be more reliable for complexes involving small partners.) We also tested the entire LBD3 sequence as well as Exon 10 in the AF3 server but did not find confident predictions of a complex with PGM1.

### Microscale-thermophoresis

For microscale-thermophoresis studies, a peptide representing the conserved Zasp motif (ZM) of ZASP/Cypher (26-mer: SSQPRQYNNPIGLYSAE*T*LREMAQMY) and a corresponding peptide with the T106I mutation (underscored in sequence) were synthesized by ABI Scientific. A vector for recombinant expression of human PGM1 has been previously described [35]. PGM1 was expressed and purified as previously described [35].

For binding studies, PGM1 was labeled using a commercial kit (NanoTemper Technologies, Inc.) according to the manufacturer’s specifications. Briefly, PGM1 was incubated at room temperature for 30 min with RED-tris-NTA dye, which specifically labels His_6_ tags, resulting in 100 nM labeled protein. The wild-type and T106I ZM peptides were serially diluted into a series of 16 PCR strip tubes and mixed 1:1 with labeled PGM1. The final concentration series of each peptide was 375 mM to 11.4 nM, with all tubes containing 50 nM labeled PGM1. Samples were incubated in the dark for 10 min prior to use. Standard capillaries (Nanotemper MO-K022) were used to draw up the liquid and placed onto the sample tray of the Monolith NT.115 instrument (NanoTemper). Measurements were performed at 22 °C with control software at 40% excitation power and 40% MST power. K_d_ values were calculated using the MO. Affinity software v.2.2.4 (NanoTemper Technologies).

### EMUDRA in silico drug repurposing

Ensemble of Multiple Drug Repositioning Approaches (EMUDRA) [36] algorithm was applied to the proteomics data using R (Version 4.3) an R-studio (V2023.09.1 + 494). For EMUDRA Fig. 8A, the significantly overexpressed proteins were used as input (log2foldchange > 0.25 and p-value 0.05). For EMUDRA Fig. 8B, the significantly decreased proteins were used as input (log2foldchange < 0.25 and p-value 0.05). The following packages were used to create the graphs: ggplot2 (3.5.1), EMUDRA (1.0.5) and EMUDRA data (1.5).

### Statistical analysis and visualization

GraphPad prism 10 for MacBook was used for the statistical analysis and violin plot generation, unless otherwise specified. Unpaired t-test with unequal group variance was used to analyse proteomics and glycopeptide data (False Discovery Rate, FDR < 0.05). Volcano plots were generated in R, using ggplot2 package. Mitocarta proteins were also analysed by Fisher’s exact test and Kruskal-Wallis (multiple t-test Dunn’s multiple comparisons test). For tracer metabolomics data, unpaired t-test with unequal group variance was performed for the metabolite abundance and fractional contribution. Two-way ANOVA with multiple t-test and Sidak correction was performed for positional labelling. Further, pathway analysis was performed with Metaboanalyst with FDR < 0.05 based on the program specifications [30]. For Seahorse oxymetry, Shapiro-Wilk test was used to determine population normality/lognormality, and multiple unpaired either parametric or non-parametric T-tests were performed with a FDR of 1%. Two-way ANOVA was performed to analyse sources of ATP. Means are represented with SD. Biorender with licence was used to generate parts of the figures. Figures were further prepared using GraphPad, MetaboAnalyst, Travis Pies [29], and Adobe Illustrator. The raw data and the details of statistical analysis for each experiment can be found in Additional Spreadsheet.

## Results

### Clinical presentation of PGM1-CDG patients included in the study

The fibroblasts of four previously reported unrelated individuals affected with PGM1-CDG were used to generate iCMs for this study. The demographic, genetic and clinical data of the four individuals with PGM1-CDG are listed in Table 1. All four individuals underwent genetic sequencing. All of the patients had different pathogenic variants in *PGM1* affecting both PGM1 isoforms (PGM1-1, PGM1-2) [9], no other variants in other genes were found. All the patients were unrelated to each other, ensuring any confounding background genetic effects were minimized.

In our cohort, three out of four patients presented with cardiac involvement. The oldest reported PGM1-CDG patients were 49 [37] and 53-years-old [8] at the time of the first symptoms. Therefore, yearly monitoring of cardiac function has been recommended in all patients affected by PGM1-CDG [1]. (P2-4). The youngest individual (P1), a three-year-old female, did not present yet with cardiac involvement at the time of PGM1-CDG diagnosis. However, based on her molecular findings and her young age, this individual is likely to develop cardiomyopathy [9]. P2 had severe cardiomyopathy (ejection fraction < 10%). He suffered a cardiac arrest and was deceased at the age of 10. P3, a 16-year-old female, presented with left ventricular hypertrophy. P4 is a 20-year-old female who also presented with severe cardiomyopathy (fractional shortening 7%) and suffered from cardiac arrest but was resuscitated.

**Table 1:** Characteristics of the four individuals with PGM1-CDG whose fibroblasts were used to generate iCMs

ID	Age*/Gender	*PGM1* variants^#^	PGM1 amino acid change	Cardiac presentation
**P1 **[5, 13, 38]	3/F	c.551delT HOM	p.Phe184Serfs*9	No cardiomyopathy at the time of assessment
**P2 **[5, 13, 38]	10/M	c.1162G > A c.1547T > C	p.Glu388Lys p.Leu516Pro	CM-severe FS < 10%. Cardiac arrest- deceased
**P3 **[5]	16/F	c.1508G > A HOM	p.Arg503Gln	Left ventricular hypertrophy
**P4 **[5]	20/F	c.1145-222G > T HOM	p.Gly382ValfsTer23	Severe cardiomyopathy FS 7%, cardiac arrest- resuscitated

*age at the time of the first publication, #variants reported in the first publication.

### Differentiation of PGM1-deficient hiPSC into iCardiomyocytes (iCMs)

The successful generation hIPSC from the fibroblasts of four individuals with PGM1-CDG was confirmed by quality control tests including karyotyping. Mycoplasma testing was also performed (see methods for details). The pluripotency markers and the ability of the hiPSC to differentiate into three germ layers were assessed (Fig. 1B**)**. The PGM1-CDG (*n* = 4) and control (CTR, *n* = 4) hiPSC were differentiated into iCMs by using previously reported chemically defined protocol [20, 21] (see methods for details). The medium supplement used to differentiate and maintain iCMs also contains galactose (exact concentration proprietary information of the manufacturer, in-house estimate 0.5mM). The schematic diagram demonstrating the main steps of the protocol used for the direct differentiation of hiPSC into to iCM is shown in Fig. 1C. Lactate selection was performed to remove any cell types that were not cardiomyocytes, such as fibroblasts as described in the method section. Successful differentiation of hiPSC into iCM was confirmed by assessing cardiomyocyte markers [39] by gene expression analysis, myosin light chain 7 (*MYL7*), myosin heavy chain 7 (*MYH7*), and troponin *(cTNT)* (Fig. 1D) and immunohistochemistry (connexin-43, alpha-actinin, cardiac troponin) **(**Fig. 1E).

### PGM1-deficient iCM exhibit decreased beating frequency and contractility

iCMs generate spontaneous extracellular field potentials that recapitulate cardiomyocyte beating in vitro and can be quantitatively assessed by multi-electrode array (MEA) recordings (Fig. 1F) [21, 40, 41]. During differentiation, PGM1-deficient iCMs showed reduced yield and delayed onset of spontaneous beating, with some cultures failing to beat. MEA analysis revealed a significant reduction in beating frequency in PGM1-deficient iCMs compared with controls (Fig. 1G), consistent with the cardiac dysfunction reported in individuals with PGM1-CDG. Representative MEA traces are shown in Fig. 1H. In addition, PGM1-deficient iCMs exhibited markedly reduced contractility (Fig. 1I) that was not rescued by electrical pacing (0.3-ms pulses, 1200 mV, 0.5–1.0 Hz; Fig. 1J). Contraction kinetics were significantly prolonged, with delayed initiation, slower time to peak contraction, and extended relaxation phases (Fig. 1K), indicating impaired excitation–contraction coupling and a bradycardic, hypo-contractile phenotype.

#### Sarcomeric protein dysregulation and potential Z-disk impairment in PGM1-deficient iCMs

As PGM1 iCMs already displayed severe dysfunction during the differentiation, we sought to investigate the consequences of PGM1 deficiency on the proteome level. We first performed a multiplexed proteomic analysis in PGM1-deficient and healthy CTR iCMs and identified 7751 unique proteins across all samples and found profound changes in global protein expression in PGM1-deficient iCMs (Fig. 2A, Ad. Fig. 1). PGM1 was among the top significantly downregulated proteins (average FC = 0.48) in all PGM1-deficient iCMs as compared to CTR (Fig. 2B), confirming the deleterious effect of *PGM1* pathogenic variants found in patients (Table 1). Consistent with cardiac dysfunction observed with MEA, several proteins involved in cardiomyocyte function and sarcomere formation were significantly decreased in PGM1-deficient iCMs such as the proteins related to sarcomere structure such as DMD, XIRP1, TNN1, TNNI1, TNNT2, ACTN2, MYOZ2 (Fig. 2A, B). Proteins involved in calcium handling and excitation–contraction coupling were also altered (Additional Fig. 2). Specifically, components of the voltage-gated calcium channel complex and the ryanodine receptor pathway were detected in both CTR and PGM1-deficient iCMs, with RyR2 representing the predominant ryanodine receptor isoform. Notably, TNNT2, which links cytosolic calcium signaling to actin–myosin crossbridge formation, was significantly reduced in PGM1-deficient iCMs (Fig. 2; Ad. Fig. 2), providing a molecular correlate for the delayed contraction kinetics observed functionally (Fig. 1).

In contrast to the depletion of sarcomeric and excitation–contraction proteins, several extracellular matrix (ECM) and collagen-associated proteins, including IGFBP7, TGFBI, COL5A1, and COL11A1, were significantly increased, suggesting a shift toward maladaptive structural remodeling. Proteins involved in cell-cycle regulation and mitochondrial function were concurrently reduced (Fig. 2A; Ad. Fig 1), indicating broader perturbations in cardiomyocyte homeostasis. Previous histological studies in a cardiac-specific PGM1 knockout mouse revealed Z-disk disarray accompanied by swollen and fragmented mitochondria [18]. Given earlier reports demonstrating colocalization of PGM1 with the sarcomeric Z-disk protein LDB3 (ZASP/Cypher) in rat cardiomyocytes and the role of pathogenic *LDB3* variants in dilated cardiomyopathy [16], we were specifically interested in further assessing the possible relationship of PGM1 with ZASP/Cypher in iCMs. LDB3 protein abundance was significantly reduced (average fold change = 0.69; Fig. 2B), along with the key interacting partners within the Z-disk ACTN2 and MYOZ2 [17], suggesting compromised Z-disk integrity.

To directly test whether PGM1 interacts with LDB3 in a manner relevant to cardiac pathology, we performed in silico structural modeling using AlphaFold3 [34]. This analysis identified a moderate-confidence interaction between PGM1 and the cardiac-specific exon 4 (ZASP motif, ZM) of LDB3 (Fig. 2C-F). Importantly, in vitro binding assays showed high-affinity binding (low µM) between PGM1 and the wild-type ZM peptide, whereas a dilated cardiomyopathy-associated *LDB3* T206I mutant [42] peptide substantially reduced binding affinity (high µM) (Fig. 2G). These experiments provide the first direct biochemical evidence that PGM1 binds a cardiac-specific region of LDB3 and that disease-associated disruption of this interaction weakens PGM1–LDB3 coupling.

Together with previous reports [16], these findings support a model in which PGM1 functions as an integral component of the LDB3-associated Z-disk network in cardiomyocytes. The in vitro binding of PGM1 to a cardiac-specific LDB3 [42] isoform provides a mechanistic explanation for the cardiac selectivity of PGM1 deficiency and suggests that disruption of Z-disk integrity directly contributes to cardiomyopathy in PGM1-CDG.

### PGM1-deficient iCMs present with mitochondrial dysfunction

Sarcomeres are organized energy-demanding structures whose contractile function depends on the close spatial and functional coupling between the myofibrillar apparatus and mitochondria. Beyond serving as ATP generators, mitochondria have recently been implicated as active contributors to sarcomere organization and stability [43]. Considering the importance of mitochondria and their proximity to sarcomere [44], it is not surprising that the cardiomyocyte architecture disruption has already been linked to mitochondrial dysfunction [45, 46]. Particularly, pathogenic variants in sarcomeric genes, including *LDB3*, were shown to also result in mitochondrial abnormalities [42, 47–49]. Analogously, mitochondrial abnormalities have previously been shown using histology in a heart of a PGM1-CDG patient, who underwent transplantation [18], as well as in a cardiac-specific PGM1 KO mouse [18].

**Fig. 3:**
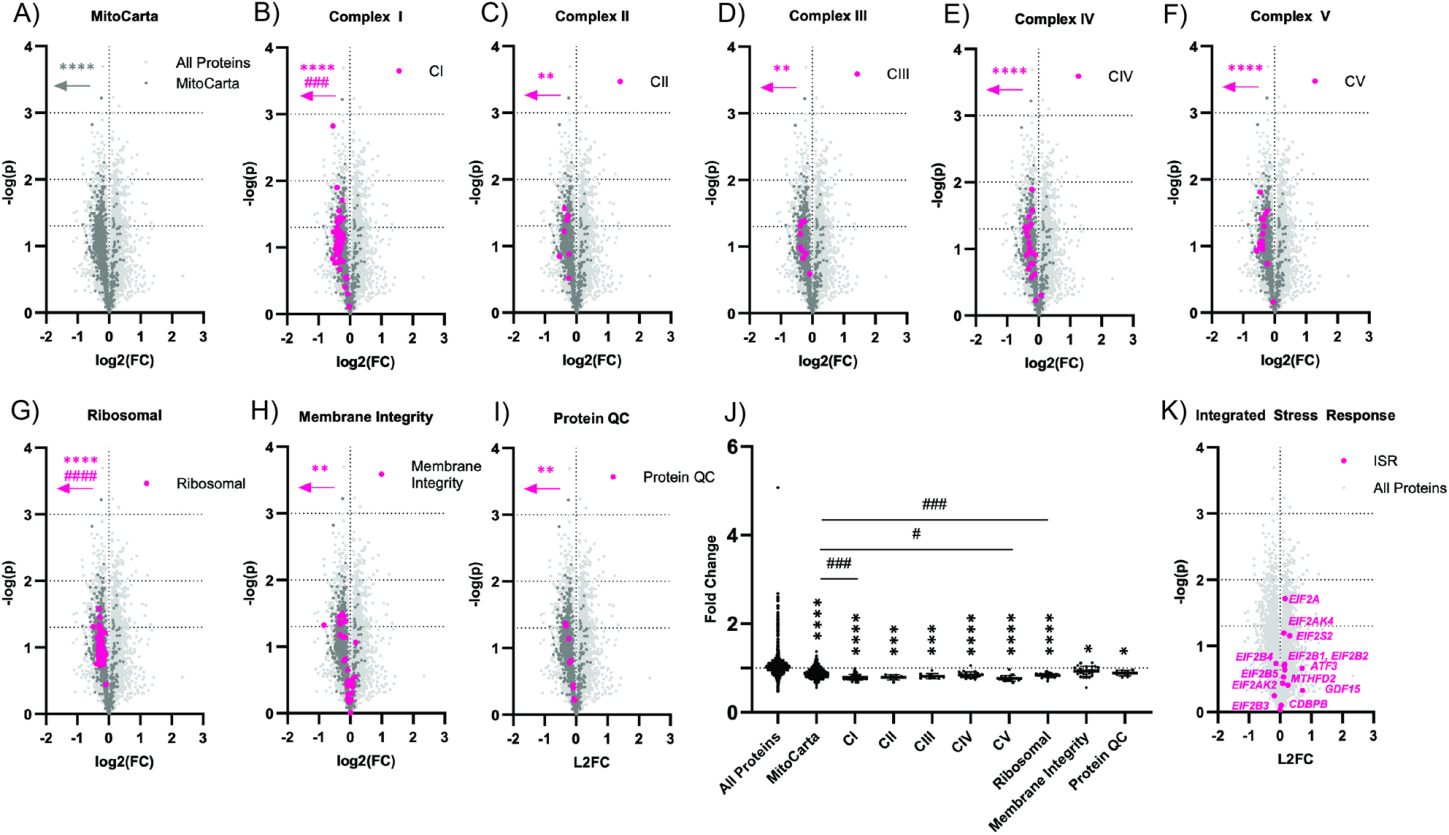
Mitochondrial proteins are depleted in PGM1-deficient iCMs. [16] **A**-**I**) Volcano plots displaying log2 fold change (FC) vs. -log p value (p) of all proteins **A**), MitoCarta proteins **A**), and the subunits of complex I **B**), complex II **C**), complex III **D**), complex IV **E**), complex V **F**), and the mitochondrial ribosome **G**) and all proteins associated with mitochondrial membrane integrity **H**) and protein quality control (QC) **I**) in PGM1-CDG cardiomyocytes (iCMs) relative to controls. Arrows indicate an increased number of depleted proteins species (Fisher’s exact test) of each subunit pool relative to the total protein pool (*) or the MitoCarta protein pool (#). **J**) Scatter plot of fold changes of all proteins, MitoCarta proteins, and the subunits of CI, CII, CIII, CIV, CV, and the mitochondrial ribosome, as well as proteins involved in mitochondrial membrane integrity and protein quality control in PGM1-CDG iCMs relative to controls. Significantly reduced average fold change (Kruskal-Wallis test) relative to all proteins (*) and the MitoCarta protein pool (#) are indicated. **K**) Volcano plots displaying log2(FC) vs. -log(p) of integrated stress response proteins in PGM1-CDG iCMs relative to controls. (#/*) *p* < 0.05, (##/**) *p* < 0.01, (###/***) *p* < 0.001, (####/****) *p* < 0.0001

To understand the extent of the mito-proteome changes in PGM1-deficient iCMs, we compared the proteomics findings with the MitoCarta protein pool, a catalogue of over 1000 genes encoding the mammalian mitochondrial proteome [23] (Fig. 3A) (see methods for details). PGM1-CDG iCM’s displayed a profound depletion in the MitoCarta protein pool, with 84% of the 912 MitoCarta proteins identified displaying a fold change < 1, compared to the 47% of the total protein pool displaying a fold change < 1 relative to controls (Fig. 3A), and an average depletion of 13% relative to the total protein pool (Fig. 3J). Consistent with this depletion of the MitoCarta protein pool, we also observed significant depletions of the subunits of CI, CII, CIII, CIV, CV, and the mitochondrial ribosome (Fig. 3B-G), with average depletions of 22%, 22%, 20%, 17%, 24%, and 18%, respectively (Fig. 3J). Additionally, the subunits of CI and the mitochondrial ribosome displayed even further depletion relative to the already depleted MitoCarta protein pool (Fig. 3B, G), with average depletions of 11% and 6% relative to the MitoCarta protein pool respectively (Fig. 3J).

As mitochondrial structural abnormalities were found in the PGM1-CDG patient’s heart and cardiac-specific PGM1 KO mouse model [18], we assessed the expression of the proteins involved in mitochondrial membrane integrity, biogenesis/remodeling, protein import and protein quality control, as well as proteins involved in mitochondrial dynamics/interorganelle communication [24] (Fig. 3B). Proteins associated with mitochondrial membrane integrity displayed significant depletions relative to the total protein pool (Fig. 2H), with an average depletion of 9% (Fig. 3H). Within this subset of membrane integrity-associated proteins, proteins associated with protein quality control (QC) were also depleted (Fig. 3I), with an average depletion of 13% relative to the total protein pool (Fig. 3J). These results further support the previously reported mitochondrial structural abnormalities in the PGM1-deficient heart [18].

Given the profound depletion in the total MitoCarta protein pool (Fig. 3B), we hypothesized that the integrated stress response (ISR) pathway may be activated in PGM1-CDG iCMs. The ISR is activated in response to cell stress, including metabolic cell stress [50]. Activation of the ISR results in phosphorylation and subsequent sequestration of Eukaryotic Transcription Initiation Factor 2 Subunit Alpha (eIF2a), which is required for initiation of protein translation, reducing global protein translation. Activation of the ISR also induces the expression of multiple genes involved in alleviating cell stress, including AT3, ATF4, ATF 5, GDF15, FGF21, MTHFD2, and CDBPB. Relatively few components of the integrated stress response pathway were identified through proteomic analysis, including eIF2a, multiple eIF2 kinases, several subunits of eIF2B, as well as multiple downstream protein species, viz. ATF3, GDF15, MTHFD2, and CDBPB. Of these, only eIF2a displayed significant enrichment (12%, *p* = 0.019) (Fig. 3K). While the phosphorylation status of this enriched eIF2A could not be assessed, our proteomic data did not reveal a systemic upregulation of key ISR markers, suggesting it may not be the primary stress response pathway activated in PGM1-deficient iCMs.

Taken together, and consistent with histological evidence from PGM1 patient tissue and cardiac-specific PGM1 KO [18], these data suggest that PGM1 deficiency in human cardiomyocytes is associated with severe and coordinated mitochondrial proteome remodeling. Importantly, the nature and extent of these mitochondrial changes parallel those reported in LDB3-related cardiomyopathy [47–49], supporting a mechanistic link between sarcomeric disruption and mitochondrial dysfunction downstream of PGM1 loss.

#### Pathway analysis highlights mitochondrial dysfunction as the major hallmark in PGM1-deficient iCMs

**Fig. 4:**
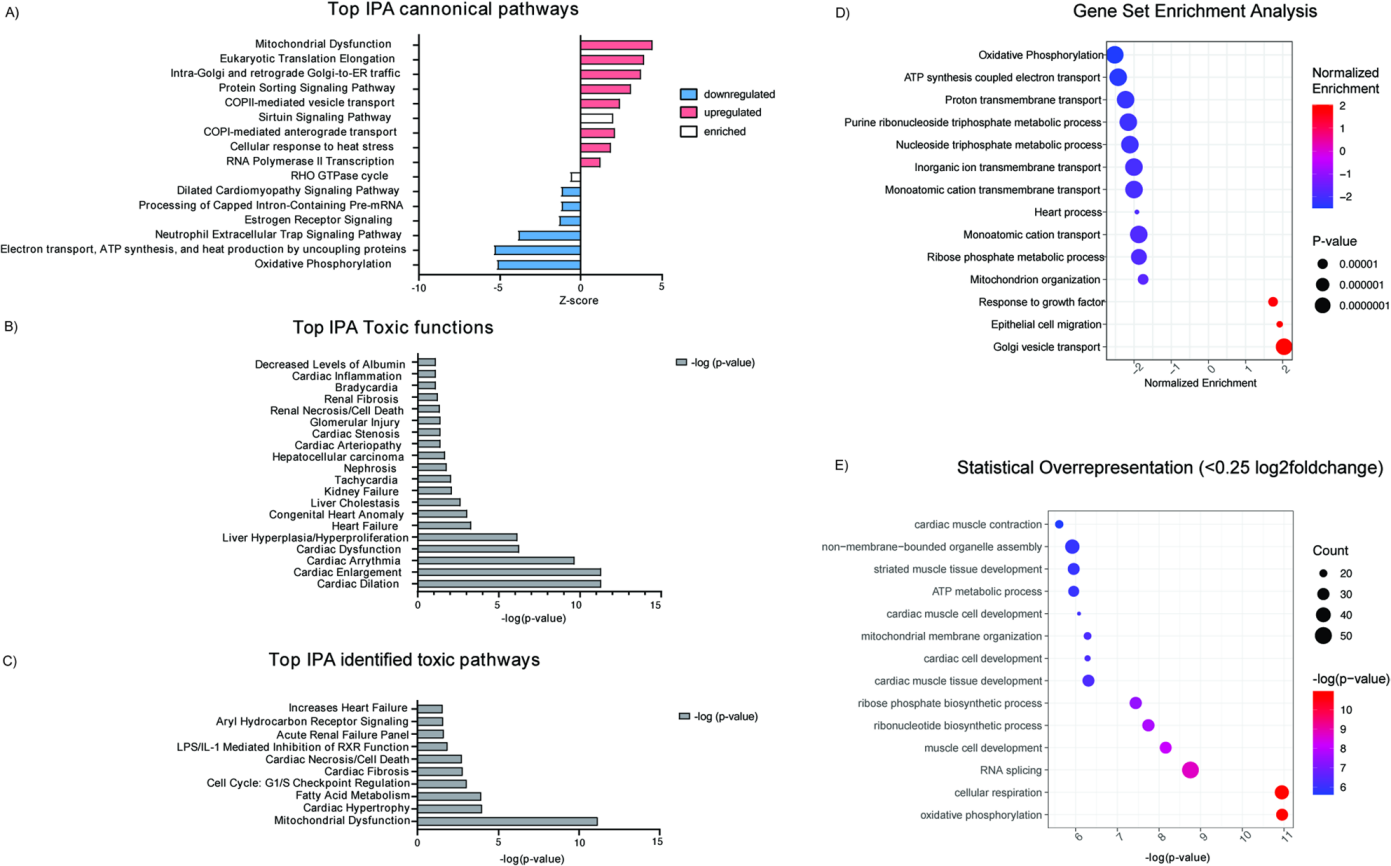
Pathway analysis of the significant proteomic changes in PGM1-deficient iCM. **A**) Top changing canonical pathways according to Ingenuity Pathway Analysis (IPA) The pink color represents upregulated pathways, while blue color signifies downregulated pathways. White color represents pathways that are enriched in the dataset, but the direction of their activity (upregulated/downregulated) cannot be predicted. **B**) IPA identified top significant toxic functions associated with the proteomic signature of PGM1-deficient iCM. **C**) IPA identified top significant toxic pathways based on the significant proteomics changes in the PGM1-deficient iCM **D**) GSEA results based on pre-ranked proteome log2foldchange- and p-values of proteomics data of PGM1-deficient iCMs compared to healthy controls. As pathways, all Biological Process (BP) pathways of the GO ontology were included. The graphs show pathways with p-value  < 0.0001 for downregulated pathways (corrected using Benjamin Hochberg method) and *p*-values  < 0.001 for upregulated pathways. (PGM1 n = 3, t = 1; CTR n = 4, t = 1)

To avoid bias in assuming that the disruption of cardiac cytoskeleton, including sarcomere and mitochondria, were the major hallmarks of PGM1-deficient heart, we employed Ingenuity Pathway Analysis (IPA, QIAGEN, see methods) to independently assess the extent of the pathways affected in PGM1-deficient iCMs and generate any additional hypothesis (Fig. 3A, B). IPA analysis showed several canonical pathways were significantly changed in PGM1-deficient iCMs (Fig. 3A). The top downregulated pathways were associated with oxidative phosphorylation, electron transport chain, ATP production and heat production by uncoupling proteins (Fig. 4A). The top upregulated canonical pathways in PGM1- deficient iCMs were mitochondrial dysfunction, eukaryotic translation elongation, intra-GA and retrograde-GA to ER trafficking as well as protein sorting (Fig. 4A). In addition, other pathways associated with cellular trafficking were significantly affected (Fig. 4A).

Corresponding to the observed decrease of proteins involved in cardiomyocyte/sarcomere machinery (Fig. 2), the top 5 upregulated toxic functions in PGM1-deficient iCMs were associated with cardiac dilation, cardiac enlargement, cardiac arrythmia, cardiac dysfunction and heart failure (Fig. 4B**).** We also used IPA to search for the toxic (disease-causing) pathways that overlap with the signature of PGM1-deficient iCMs. The top toxic pathways associated with PGM1-deficient proteome were mitochondrial dysfunction and cardiac hypertrophy (Fig. 4C).

In addition, we performed gene set enrichment analysis (GSEA) [51]. GSEA showed several significantly enriched pathways (Fig. 4D,E,F). Downregulated pathways included oxidative phosphorylation, purine/nucleotide, ribose phosphate metabolism and inorganic ion transport. Upregulated pathways included response to TGF-beta, collagen and ECM pathways (Fig. 4E, F).

Together, these unbiased pathway analyses converge on mitochondrial dysfunction, altered nucleotide metabolism, and extracellular matrix remodeling as the dominant molecular signatures of PGM1-deficient iCMs, providing a systems-level framework that links sarcomeric destabilization to metabolic failure and structural remodeling as key contributors to the severe cardiac phenotype observed in PGM1-CDG.

#### Glucose flux is altered in PGM1-deficient iCMs

**Fig. 5:**
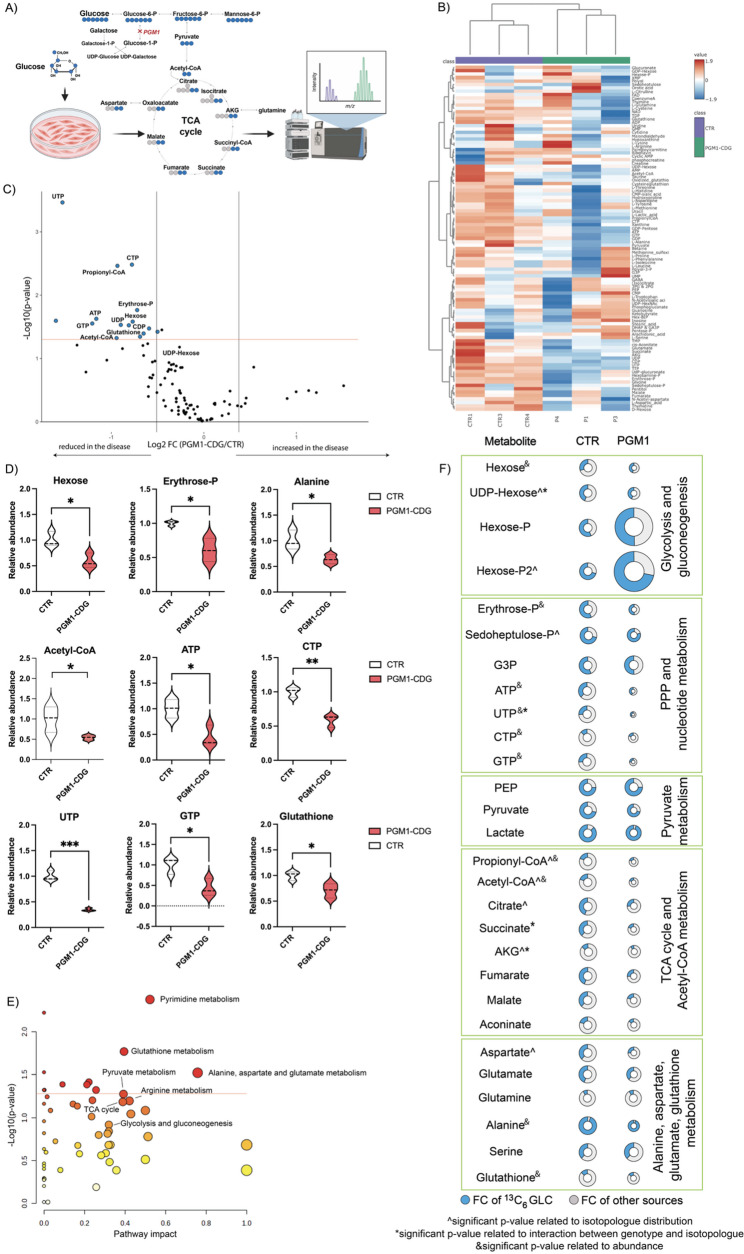
Glucose flux is altered in PGM1-deficient iCMs.** A**) Schematic representation of the tracer metabolomics methodology. ^13^C_6_-glucose is added to the medium not containing glucose (but containing ^12^C_6_-galactose).** B**) Volcano plot showing the most significantly changing metabolites (FC < 0.7, p-value < 0.05) in PGM1-deficient iCMs. **C**) Heatmap of all relatively quantified metabolites in CTR and PGM1-deficient iCMs. **D**) Violin plots showing the most significant metabolite changes in PGM1-deficient iCMs compared to CTR. Significance is indicated as * *p* < 0.05, ** *p* < 0.01, *** *p* < 0.001. **E**) Pathway analysis with top identified changing pathways in PGM1-deficient iCMs. FDR < 0.05 was performed (see methods). **F**) Schematic representation of the metabolic consequences of PGM1 deficiency and altered glucose flux in metabolites identified by tracer metabolomics analyses. Significant differences in isotopologues (positional) labeling of the metabolites were assessed by TWO-way ANOVA. Specific changes in positional labeling are represented in Sup Fig. 2 and Sup Fig. 3. Significant differences in labeling related to either genotype or interaction between genotype are indicated either **^** or * respectively. The significant differences in the abundance of the metabolites are indicated with a &. PGM1 *n* = 3, t = 2–3; CTR *n* = 3, t = 2–3. Violin plots are shown with standard deviation (SD). Detailed statistical analysis can be found in the supplemental information

To further define how PGM1 deficiency affects cardiac metabolic homeostasis and mitochondrial energy production, we performed targeted tracer-based metabolomics with ^13^C_6_-glucose focusing on central carbon metabolism (Fig. 5A). As mentioned above, the medium used to differentiate iCMs already contains galactose (exact concentration not provided as its manufacturer’s proprietary information, in-house estimate 0.5 mM), allowing us to partially assess the changes independent of glycosylation. PGM1-deficient iCMs exhibited a broad reduction in glycolytic and anaplerotic intermediates, including hexoses (glucose, galactose, mannose, and fructose), erythrose-4-phosphate, alanine, and the redox-related metabolite glutathione (Fig. 5B, C, D). In parallel, levels of propionyl-CoA and high-energy nucleotide triphosphates (ATP, GTP, CTP, and UTP) were significantly decreased, consistent with impaired mitochondrial-supported bioenergetic capacity. In contrast, UDP-hexose levels were maintained in PGM1-deficient iCMs at levels comparable to controls (Fig. 5B), consistent with prior observations that galactose increases the UDP-hexose pool and improves glycosylation in PGM1-CDG, and with the presence of galactose in the iCM differentiation medium (manufacturer proprietary; in-house estimate ~ 0.5 mM).

To visualize the most affected pathways in PGM1-deficient iCMs, we used pathway analysis MetaboAnalyst tool (see methods, Additional data) [30]. The most significantly affected metabolic pathways included pyrimidine; glutathione; alanine, aspartate and glutamate metabolism as well as TCA cycle; arginine and pyruvate metabolism (Fig. 5D, E), all strongly linked to mitochondrial function. Finally, the fractional contribution (FC) and isotopologues labeling of ^13^C_6_-glucose showed significant metabolic rewiring in PGM1-deficient iCMs resulting in an altered glucose flux through metabolites of pentose phosphate pathway (PPP), nucleotide synthesis, and TCA cycle (Fig. 5E, Ad. Figs. 3 and 4). These results indicate that even in the presence of galactose, PGM1-deficient iCMs show a suppression of central carbon metabolism downstream of TCA cycle, supporting the hypothesis that mitochondrial function is disrupted, regardless of glycosylation.

#### PGM1-deficient iCM have reduced mitochondrial respiratory flux

**Fig. 6:**
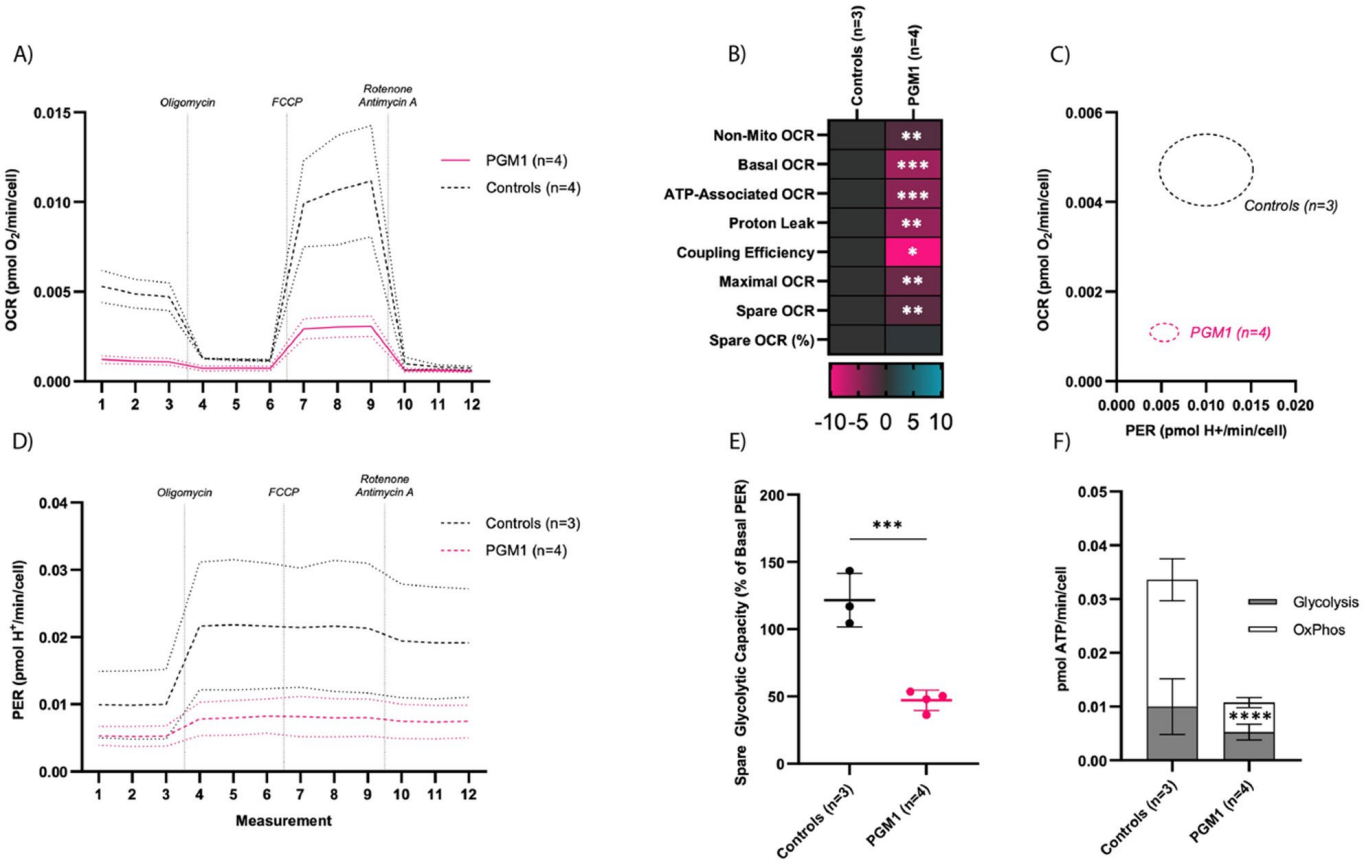
PGM1-deficient iCM show decreased basal, maximal and ATP-linked respiration. Mitochondrial stress test normalized to cell count. (**A**) Oxymetry plot displaying Oxygen Consumption Rate (OCR) of PGM1 and Control iCMs (mean +/- SD) during Mito Stress Test Assay. (**B**) Heat map of Mito Stress Test OCR readouts, representing SDs from the mean of the controls. (**C**) Metabolic map displaying Proton Efflux Rate (PER, readout of ECAR) on the X axis and OCR on the Y axis. Circles represent SD of the means of each group. (**D**) Proton efflux rate (PER) plot displaying PER of PGM1 and Control iCMs (mean +/- SD) during Mito Stress Test Assay. (**E**) Spare glycolytic capacity. (**F**) Bar plot displaying sources of ATP. **B**) Mitochondrial stress test normalized to CS. The P values are indicated as significant * (< 0.05), ** (< 0.01), or *** (< 0.001). (PGM1 *n* = 4, t = 3 CTR *n* = 3, t = 3)

To directly interrogate mitochondrial respiratory capacity and glycolytic flux in PGM1-deficient cardiomyocytes, we performed extracellular flux analysis using the Seahorse XFe96 platform (Agilent). To reduce methodological artefacts, the cells were maintained in the same medium used in the previous experiments and the cell viability and cell count were assessed before and after the experiments (see methods). Both healthy CTR and PGM1-deficient iCMs were plated at the same time, on the same plate, and several technical replicates were used (n = 3-4). The experiments were repeated 3 times. PGM1-deficient iCMs displayed reduced oxygen consumption rate (OCR) relative to cell number (proxy for DNA and protein content) (Fig. 6A) at all time points of the Cell Mito Stress Test assay. PGM1-CDG iCMs also displayed significantly reduced non-mitochondrial respiration, basal respiration, ATP-associated respiration, proton leak, coupling efficiency, maximal respiration, and spare OCR relative to controls (Fig. 6B). No difference in proton efflux rate (PER, readout of external acidification rate- ECAR) was observed (Fig. 6D). The metabolic map of resting OCR and PER showed PGM1 iCMs clustered separately from the controls, characterized by greatly reduced OCR and only moderately reduced PER (Fig. 6C). While basal PER was not significantly reduced in PGM1-CDG iCMs, spare glycolytic capacity (the change in PER following oligomycin administration) as a percentage of basal PER was highly significantly reduced relative to controls (Fig. 6E). Based on the OCR and PER data derived using the Cell Mito Stress Test kit, pmol of ATP produced through oxidative phosphorylation (OxPhos) and glycolysis were calculated according to the manufacturer instructions. PGM1-CDG iCMs displayed a highly significantly reduced total ATP production, as well as a highly significantly reduced OxPhos-derived ATP production (Fig. 6F), while glycolysis-derived ATP-production was not significantly affected. Consistent with our tracer metabolomics findings, these data suggest PGM1-deficient iCMs do not exhibit a compensatory increase in glycolysis despite markedly reduced mitochondrial respiration.

As the differences in respiration can also be driven by the changes in mitochondrial mass/abundance, we also normalized the data to citrate synthase (CS) activity, which is a proxy readout for mitochondrial mass/abundance. We found no significant difference in CS activity between PGM1-deficient and CTR iCMs (Add. Fig. 5). Normalized to CS, the proton leak, maximal respiration, and basal respiration were significantly decreased in PGM1-deficient iCMs, as was the spare glycolytic capacity and the pmols of ATP being derived from OxPhos (Add. Fig. 5). These findings indicate that PGM1-deficient iCMs exhibit severely reduced mitochondrial respiration, primarily driven by alterations in mitochondrial ATP-linked respiration.

### Glycosylation in PGM1-deficient iCMs

**Fig. 7:**
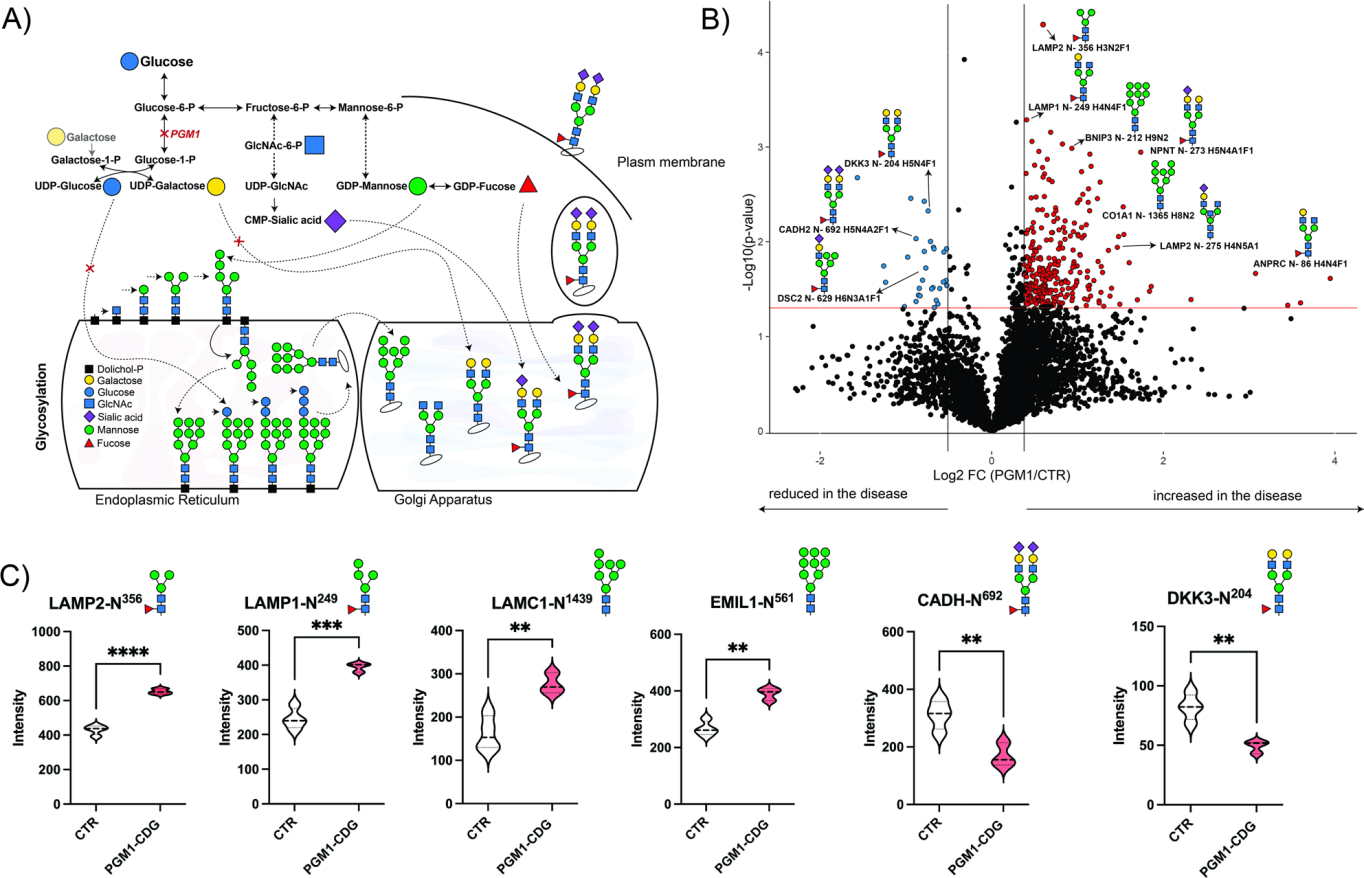
Global glycoproteomic changes in PGM1-deficient iCMs (iCM). **A)** Schematic representation of PGM1 in relation to glycosylation. Glycosylation starts in cytosol, where suger nucleotides (UDP-Glucose, UDP-Galactose, UDP-GlcNAc, GDP-Mannose, GDP-Fucose, CMP-sialic acid etc.) are produced. The sugar nucleotides serve as donors of sugars to the growing glycan chains in Endoplasmic reticulum (ER), where glycans are attached to proteins and then further processed in Golgi apparatus (GA). Fully formed glycans are exported to the cell surface. **B**) Volcano plot showing differentially abundant glycoproteins in PGM1-deficient iCM compared to healthy control iCM (CTR) after correction for the proteins that were significantly changing on protein level. X-axis shows log_2_ fold change (FC) (PGM1-deficient/CTR) and Y-axis represents the -log10 of p-value. The horizontal red line represents the cutoff for significance [-log10(0.05)]. The most significantly changing proteins are marked in either red (> 1.3 FC, *p* < 0.05) or blue (< 0.7, *p* < 0.05). **C**) Violin plots showing some of the top significantly changing glycoproteins in PGM1-deficient iCM compared to CTR. Y-axis represents the ion intensity of TMT channels. PGM1 *n* = 3, t = 1; CTR *n* = 4, t = 1. Violin plots are shown with standard deviation (SD). Detailed statistical analysis can be found in the supplemental information

Apart from its role in metabolism, PGM1 plays a central role in protein glycosylation, and PGM1 deficiency classically results in glycosylation abnormalities due to depletion of the UDP-hexose pool (Fig. 7A), a defect that can be partially corrected by galactose supplementation (Fig. 7A) [10]. Importantly, the medium used to culture iCMs contains galactose (0.5mM) and tracer metabolomics experiments showed UDP-hexose pool was not depleted compared to CTR iCMs (Fig. 5). Despite this preservation of glycosylation substrate availability (galactose), untargeted proteomics showed marked depletion of proteins essential for cardiomyocyte architecture and mitochondrial function, and functional assays confirmed profound mitochondrial metabolic and respiratory impairment in PGM1-deficient iCMs (Figs. 2, 3, 5 and 6). Given that the vast majority of sarcomeric and mitochondrial proteins are not glycosylated, these observations suggest that galactose is unlikely to directly rescue the structural and bioenergetic defects observed in PGM1-deficient iCMs. To assess the glycosylation status in PGM1-deficient iCMs in presence of galactose and further test our hypothesis, we performed untargeted glycoproteomics in PGM1-deficient and CTR iCMs. Untargeted glycoproteomics integrates proteomics, which assesses changes of proteins, and glycomics, which assesses different glycans attached to the proteins, allowing us to understand the structure, function, and variations of these glycoproteins.

Using this method, we identified 4772 unique glycopeptides derived from 574 proteins across all samples (CTR and PGM1). We found several changes in glycosylation in PGM1-deficient iCMs compared to CTR, out of which the abundances of 486 unique glycopeptides were significantly changing (*p* < 0.05) (Additional Data). As we identified significant changes in the proteome of PGM1-deficient iCMs (Fig. 2, Additional Data), we then performed additional analysis, by filtering out the glycopeptides coming from proteins whose abundance was also significantly changed at the protein level (*n* = 46) resulting into 377 significantly changing glycopeptides (Fig. 7B). Most of them belonged to ECM (e.g. NPNT, CO5A2, EMIL1, ALCAM), and membrane/lysosomal trafficking (LAMP1, LAMP2) and a few glycopeptides of metabolism/mitochondrial function (e.g., BNIP3, LRP1, SAP) (Additional data).

The upregulated glycoproteins (*n* = 98, FC > 1.3, *p* < 0.05) contained high mannose or paucimannose glycans (e.g. BNIP3-N^212^ H9N2, LAMP2-N^356^ H3N2F1, LAMP1-N^249^ H4N2F1, LAMC1- N^1439^ H7N2) or truncated complex glycans (e.g., LAMP1-N^249^ H5N4F1, NPNT-N^273^ H5N4A1F1) (Fig. 7B, C**)**. Similarly, only a few of the significantly changing downregulated glycoproteins (*n* = 18, FC < 0.7, *p* < 0.05) contained complex (e.g. DKK3-N^204^ H5N4F1, CADH2-N^692^ H5N4A2F1) and hybrid glycans (e.g. DSC2-N^629^ H6N3A1F1). Although not extensive, the observed increase of glycoproteins containing high mannose and truncated glycans and a decrease of complex glycans are consistent with previous glycosylation pattern seen in PGM1-deficient patients [5, 10]. Although we did not perform orthogonal glycomics or lectin-based assays to independently assess global glycosylation in PGM1-deficient iCMs, glycoproteomic profiling indicates that even low levels of galactose are sufficient to partially restore glycosylation in PGM1-deficient iCMs. In contrast, sarcomeric and mitochondrial dysfunction persist (Figs. 2, 3, 4 and 5), strongly supporting the hypothesis that galactose-resistant PGM1-deficient cardiomyopathy is driven primarily by glycosylation-independent defects in cardiomyocyte structure and bioenergetic function.

### In silico drug repurposing reveals potential drug candidates targeting cardiac development and mitochondrial function

**Fig. 8:**
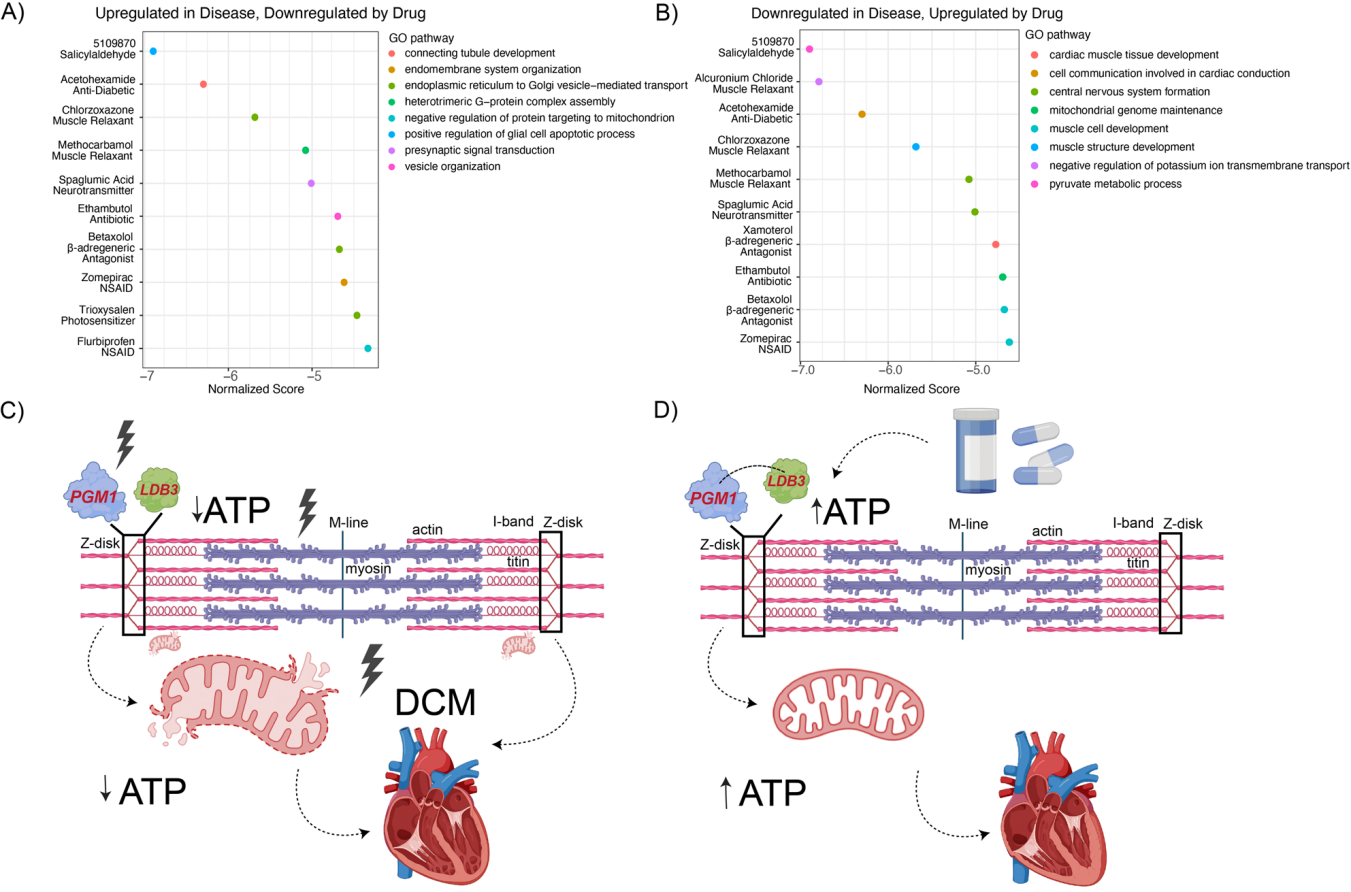
Exploring therapeutic options for cardiac presentation in PGM1 deficiency. **A**) EMUDRA identified drugs that are predicted to upregulate the processes that are downregulated in PGM1-deficient iCMs based on Differentially Expressed Gene analysis (log2foldchange < 0.25, p-value < 0.05). **B**) EMUDRA identified drugs that are predicted to downregulate the processes that are upregulated in PGM1-deficient iCMs based on Differentially Expressed Gene analysis (absolute log2foldchange > 0.25, p-value < 0.05). The top-11 drug candidates are shown for both analysis. The Drug Class is written below the CMAP drug. Normalized enrichment Scores indicate the potential of the drug to normalize the phenotype, and lower scores are associated with higher potential of normalizing aberrant log2foldchange values. **C**) potential pathomechanism linking PGM1 deficiency to sarcomere disruption and mitochondrial dysfunction, eventually resulting in cardiac failure in PGM1-CDG, **D**) potential therapeutic mechanism in PGM1 deficiency targeting sarcomeric proteins resulting in improved cardiac function

Despite substantial advances in understanding and managing mitochondrial dysfunction across a range of mitochondrial diseases, there are currently no FDA-approved therapies that directly target mitochondrial pathology [52]. Therefore, treating mitochondrial dysfunction remains a challenge. To identify potential novel cardiac-specific treatment strategies in PGM1-deficient cardiomyopathy, we used Ensemble of Multiple Drug Repositioning Approaches (EMUDRA) [36], a hypothesis generating tool, on our untargeted proteomics data. EMUDRA compares the effects of ~ 1300 FDA approved small molecules and predicts whether these molecules can effectively normalize log2foldchange values (see methods for details). This proof-of-concept analysis identified several drugs that are predicted to downregulate processes that are upregulated in PGM1-deficient iCMs, and were also reflected in the GSEA results, such as vesicle organization, ER to Golgi transport, protein targeting to mitochondria (Fig. 8A). In addition, EMUDRA identified several drugs that are predicted to upregulate processes that are downregulated in PGM1-deficient iCMs, such as mitochondrial genome maintenance, pyruvate metabolism, cardiac muscle tissue development, muscle structure and cell development, and cell communication involved in cardiac conduction (Fig. 8B). Several drugs were shown to target both upregulated and downregulated processes. These included for example NSAIDs such as zomepirac, chlorzoxazone, a muscle relaxant, betaxolol, β1selective (cardioselective) adrenergic antagonist, and compound 5109870, which was previously shown to increase *PGM1* expression in vitro (Fig. 8A, B). The candidates identified in the EMUDRA tool can further be tested in vitro to assess their therapeutic potential. Although experimental validation of these candidates lies beyond the scope of the present study, these analyses provide a starting point for future in vitro and in vivo testing of targeted therapeutic strategies for PGM1-deficient cardiomyopathy.

In summary, our findings suggest PGM1 is an important regulator of cardiac structural integrity, whose deficiency likely destabilizes the sarcomeric Z-disk through impaired interaction with LDB3 and results in mitochondrial dysfunction (Fig. 8C), independent of glycosylation. This mechanistic framework suggests that therapeutic strategies aimed at restoring sarcomeric stability and mitochondrial function could be a good strategy to effectively treat the galactose-resistant cardiomyopathy observed in PGM1-CDG (Fig. 8D).

## Discussion

PGM1 deficiency is associated with severe, often lethal cardiac complications [1, 3]. While its role in metabolism and glycosylation is well established, the structural function of PGM1 in the human heart remains unclear. Previous studies using an in vivo cardiac-specific PGM1 KO mouse model [18] as well as in vitro skeletal muscle PGM1 KO mouse model [53] implicated mitochondrial dysfunction as a potential contributor to the cardiac phenotype in PGM1-deficiency. However, the underlying pathomechanism remained unresolved, and these findings have not been translated or validated in a human disease–relevant model. Here, we tested the hypothesis that PGM1 deficiency causes cardiomyocyte dysfunction through a glycosylation-independent disruption of sarcomeric–mitochondrial coupling, leading to impaired energy metabolism and contractile failure. To test this hypothesis, we generated and characterized PGM1-deficient induced pluripotent stem cell–derived cardiomyocytes (iCMs) from four individuals harbouring five distinct pathogenic *PGM1* variants (Fig. 1B-E). The iCMs were cultured in the medium containing galactose, which allowed us to partially investigate the cardiac-specific pathomechanism behind PGM1-defiency, independent of glycosylation. Even in the presence of galactose, PGM1-deficient iCMs exhibited reduced beating capacity and impaired contractility (Fig. 1F-K), consistent with intrinsic cardiomyocyte dysfunction. To define the cardiac-specific molecular mechanisms underlying PGM1 deficiency, we then performed comprehensive multi-omics analyses in this disease-relevant human model. Untargeted proteomic analysis revealed a significant decrease in PGM1 expression across all PGM1-deficient iCMs, accompanied by upregulation of extracellular matrix (ECM) proteins and marked downregulation of key proteins involved in sarcomere integrity, cardiac and mitochondrial function (Fig. 2). Given the depletion of cardiac/sarcomeric proteins, we hypothesized that PGM1 may have a structural role in maintaining cardiac architecture. PGM1 has previously been shown to associate with the Z-disk protein Zasp/Cypher (LDB3) [16]. Pathogenic variants in *LDB3* have been shown to disrupt PGM1-LDB3 binding under stress conditions, leading to defective Z-disk formation and the development of dilated cardiomyopathy in mice [16]. As *LDB3* contains cardiac-specific exons that may serve as potential binding sites for PGM1 **(**Fig. 2), we hypothesized that pathogenic variants in *PGM1* (specifically the ones disrupting PGM1-2 isoform found in our patients) impair PGM1-LDB3 binding, leading to Z-disk destabilization and exacerbating the biochemical consequences of PGM1 deficiency in the heart. Supporting this hypothesis, we observed reduced LDB3 levels in PGM1-deficient iCMs, along with decreased expression of other key Z-disk components such as ACTN2 and MYOZ2 (Fig. 2, Additional Data). In silico binding studies using AlphaFold3 [34] further implicated exon 4 of *LDB3* as a potential interaction site for PGM1 (Fig. 2C-F). Our findings suggest that PGM1 directly binds to exon 4 of LDB3, a cardiac-specific domain critical for Z-disk integrity (ZM peptide). This interaction was predicted in silico and validated through in vitro binding assays (Fig. 2G), revealing a novel mechanism by which PGM1 contributes to sarcomeric organization and cardiomyocyte function. We propose that the loss of this PGM1-LDB3 interaction in PGM1-CDG disrupts Z-disk structure, leading to impaired excitation–contraction coupling and cardiac dysfunction. Importantly, since exon 4 is unique to the cardiac isoform of LDB3, this mechanism may also explain the tissue-specific failure of dietary galactose therapy in the heart [9, 15]. Furthermore, whilst we only assessed cardiomyocytes in this study, these results also suggest that similar mechanism might be affecting skeletal muscle cells, where the PGM1 and other LDB3 isoforms might similarly interact.

While galactose can partially restore glycosylation in other tissues, it does not compensate for the structural role of PGM1 protein itself in cardiomyocytes. Thus, we propose that PGM1 may act not only as a metabolic enzyme but also as a direct regulator of Z-disk stability and expression. The sarcomere Z-disk and mitochondrial function in the heart are closely interconnected [46, 54–56]. Intermyofibrillar scaffolds within the Z-disk serve as anchors for thin filaments, transmitting force along myofibrils while regulating their length changes and preventing membrane damage [47–57]. These scaffolds are also essential for mitochondrial localization, as mitochondria are positioned at the I-band. Disruptions in myofibrillar structure often lead to abnormal mitochondrial distribution, and cardiomyopathies, including *LDB3* deficiency, are frequently associated with mitochondrial dysfunction [47–57]. Similarly, Z-disk disarray and swollen mitochondria were previously shown in PGM1 cardiac specific KO mouse model showed and in the heart of the patient with PGM1-CDG [18].

To assess the extent of mitochondrial abnormalities in PGM1-deficient iCMs, we evaluated mitochondrial protein abundances by mapping our proteomic data to the MitoCarta database. This analysis revealed a significant depletion of mitochondrial proteins, including components of complexes I-V of the electron transport chain, as well as proteins involved in mitochondrial biogenesis and quality control (Fig. 3), corroborating the previous findings and suggesting a mechanistic link between PGM1 deficiency, Z-disk instability, and mitochondrial dysfunction.

Further, Ingenuity Pathway Analysis (IPA) highlighted several affected pathways, including mitochondrial function, ECM regulation, protein sorting, and ER-to-Golgi transport pathways (Fig. 4). Toxicity analysis revealed that the PGM1-deficient proteome overlaps with signatures of cardiac arrhythmia, dilation, and enlargement—common cardiac manifestations in PGM1-CDG (Fig. 4B). Both mitochondrial and heart disorders were strongly associated with the PGM1-deficient proteome (Fig. 4C), and the GSEA indicated mitochondrial dysfunction, cell cycle dysregulation, and ECM remodeling as affected pathways in PGM1-deficient iCMs (Fig. 4D-F).

All significantly altered processes highlighted by pathway analysis in PGM1-deficient iCMs, ECM remodeling, and mitochondrial dysfunction have already been linked to cardiac failure including dilated cardiomyopathy [54]. Both ECM upregulation [58, 59] and mitochondrial and OXPHOS dysfunctions have been linked to dilated cardiomyopathy [54, 60], the most common cardiac manifestation in PGM1-CDG [43]. Previous reports of abnormal mitochondrial architecture [18] and fibrosis [2] in individuals with PGM1-CDG [18] further support the connection between these processes and cardiac pathology.

As the majority of our untargeted approaches implicated mitochondrial function as one of the main drivers of cardiac phenotype in PGM1-CDG, we performed functional studies to assess mitochondrial and other metabolic changes in PGM1-deficient iCMs.

First, we performed targeted tracer metabolomics studies of central carbon metabolism. Previously, decreased UDP-Hexose levels were linked to reduced glycosylation in PGM1-CDG, which improve in presence of galactose [5]. Similarly, in the presence of galactose, UDP-hexose levels were not significantly reduced in PGM1-deficient iCMs compared to the CTR (Fig. 5). On the other hand, several metabolites were still significantly affected in PGM1-deficient iCMs even in the presence of galactose and ^13^C_6_-glucose tracer analysis revealed altered glucose flux through the pentose phosphate pathway (PPP), nucleotide synthesis, and the TCA cycle, highlighting metabolic shifts in PGM1-deficient iCMs (Fig. 5E, Sup. Fig. 2 and 3). Interestingly, while galactose has previously shown to normalize UDP-hexose levels in PGM1-deficient fibroblasts, significant changes in TCA cycle or nucleotide metabolism, were not previously observed in PGM1-deficient fibroblasts [5, 61], further suggesting that these changes might be cardiac-specific. In the heart, mitochondrial metabolism and nucleotides play a critical role, as ATP drives essential cellular and mechanical processes, including the actin-myosin bridging cycle. Depletion of nucleotides and ATP has been implicated in impaired cardiac mechanics and cardiac failure [62, 63]. Furthermore, TCA cycle is directly linked to oxidative phosphorylation (OXPHOS) and mitochondrial function, while disruptions in nucleotide synthesis have broader implications beyond energy metabolism, affecting cell cycle regulation. Notably, mitochondrial dysfunction is known to impair de novo pyrimidine synthesis through dihydroorotate dehydrogenase (DHODH) [64], a mitochondria-coupled enzyme. Additionally, previous studies have shown that electron transport chain (ETC) dysfunction suppresses *de novo* purine synthesis while upregulating the purine salvage pathway [65].

To further confirm mitochondrial dysfunction and ATP depletion in PGM1-deficient iCMs, we performed Seahorse respiration studies. While previously no significant decrease in mitochondrial respiration or OXPHOS enzyme activity were found in PGM1-deficient fibroblasts [5, 61], PGM1-deficient iCMs showed profound decrease of basal and ATP-linked respiration, consistent with mitochondrial dysfunction (Fig. 6, Add Fig. 4). Interestingly, untargeted proteomics showed a significant, however, moderate ~ 20% reduction in the abundance of complex subunits, which may suggest that downstream effects, such as impaired complex assembly or direct enzymatic inhibition, could be amplifying the mitochondrial phenotype, which was not observed in other models [5, 61]. Collectively, these results suggest that mitochondrial dysfunction is specific to PGM1-deficient iCMs and support the hypothesis that sarcomeric and mitochondrial dysfunction are primary drivers of PGM1-deficient cardiomyopathy.

To confirm that sacromeric and mitochondrial dysfunction in PGM1-deficient iCMs were independent of glycosylation, we performed untargeted (N)-glycoproteomics (Fig. 7). As iCMs are already cultured in the medium with galactose (approximately 0.5mM) and we found no difference in UDP-hexose levels between PGM1 and CTR iCMs, we suspected glycosylation was not extensively affected. In the presence of galactose, PGM1-deficient iCMs exhibited a few alterations in glycoprotein profiles compared to control iCMs, with upregulated glycoproteins predominantly containing high mannose/paucimannose glycans. In addition, a few of the glycoproteins with complex glycans were downregulated (Fig. 7B-C).

Interestingly, a few of the significantly affected glycoproteins were associated with extracellular matrix (ECM) remodeling and membrane trafficking (Fig. 7B, C). ECM alterations have been shown to affect signaling which could indirectly affect mitochondrial function, possibly contributing to cardiac phenotype in PGM1-CDG. Nevertheless [10, 13], the majority of sarcomeric and mitochondrial proteins are not glycosylated, and their expression independent of glycosylation, further highlighting sarcomere (and mitochondrial) disruption as drivers of therapy-resistant PGM1-deficient cardiomyopathy.

Finally, to identify therapeutic strategies with immediate clinical translation for PGM1-CDG patients with cardiac complications, we performed in silico (hypothesis driven) drug repurposing using the Ensemble of Multiple Drug Repositioning Approaches (EMUDRA) [36] (Fig. 8). EMUDRA identified several drug candidates, including antibiotics, HDAC inhibitors, and NSAIDs, which target pathways disrupted in PGM1-deficient iCMs. Among them, zomepirac, an NSAID, was predicted to downregulate ER-to-Golgi vesicle transport pathways while enhancing processes related to cardiac and muscle cell development as well as mitochondrial maintenance (Fig. 8A). However, due to the potential risks of long-term NSAID use, alternative candidates may provide safer therapeutic options. Though this falls beyond the scope of the present study, drug candidates identified by EMUDRA could be further functionally validated in iCMs, followed by preclinical testing in relevant animal models (such as PGM1 KO model described in [18]) for safety and efficacy, before advancing to early-phase clinical trials.

## Conclusion

In conclusion, this study defines a previously unrecognized, cardiac-specific role for PGM1 that extends beyond glycosylation to the regulation of sarcomeric integrity and mitochondrial function in cardiomyocytes. Although mitochondrial dysfunction is a prominent feature of PGM1-deficient iCMs, the current model does not allow us to definitively determine whether Z-disk disruption directly drives mitochondrial impairment or whether these processes occur independently. Nevertheless, when considered together with prior studies, our findings suggest that PGM1 deficiency disrupts interactions with the Z-disk protein LDB3, potentially resulting in coordinated sarcomeric disorganization and a secondary mitochondrial dysfunction that compromise energy homeostasis and contractile performance. Furthermore, our findings are consistent with clinical observations that galactose supplementation improves systemic features but fails to ameliorate cardiac and skeletal muscle involvement in individuals with PGM1-CDG [7, 10, 11, 13]. More broadly, our data illustrate how disruption of a single protein could simultaneously destabilize structural and metabolic networks in the heart, a paradigm that may extend to more common forms of cardiomyopathy characterized by coupled sarcomeric and mitochondrial defects. By revealing this metabolic-structural axis, our work suggests novel therapeutic strategies targeting energy metabolism or sarcomere stability might be beneficial to treat PGM1-CDG -related cardiomyopathy and potentially other related cardiomyopathies.

### Limitations of the study

The primary goal of this study was to define the cardiac-specific role of PGM1, with a focus on sarcomeric and mitochondrial mechanisms. Glycosylation abnormalities in PGM1-CDG and their correction by galactose have been extensively characterized [5, 10, 13], and multiple studies have shown that cardiac involvement does not respond to galactose therapy [3, 5, 7, 11, 18]. In our iCM model, differentiation and culture were performed in the presence of galactose, precluding galactose-free conditions, and thus limiting our ability to fully assess the extent of glycosylation defects in its absence [5]. iCMs differentiation was performed in the presence of 0.5mM galactose, a concentration lower than previously reported therapeutic levels used in vitro (∼2 mM) [5, 13]. Despite this, using untargeted glycoproteomics, we detected relatively few significantly altered glycoproteins, suggesting that even low galactose supplementation partially restores glycosylation in PGM1-deficient iCMs. Assessment of glycosylation in PGM1-iCMs under galactose-free conditions was not feasible, as galactose withdrawal would disrupt the differentiation protocol and impair cardiomyocyte maturation. Future studies incorporating complementary global glycomics approaches, including PNGase F–released glycan profiling or lectin-based analyses, may enable more comprehensive evaluation of glycosylation in PGM1-deficient iCMs. Nevertheless, PGM1-deficient iCMs exhibited profound sarcomeric and mitochondrial (protein) abnormalities despite galactose exposure, supporting a glycosylation-independent mechanism underlying cardiomyopathy. Consistent with this interpretation, in silico drug-repurposing analyses (Fig. 8) identified candidate therapies with the potential to target these defects independently of glycosylation, although experimental validation will be required.

A further limitation is that PGM1 enzymatic activity and its structural role cannot be experimentally uncoupled. While purified PGM1 and LDB3 interact in vitro independently of enzymatic activity, genetic loss of *PGM1* simultaneously affects protein abundance and catalytic function, mirroring the patient condition. To our knowledge, no tools exist to selectively inhibit PGM1 enzymatic activity without altering protein structure, limiting mechanistic dissection of these dual functions in cardiomyocytes. At present, the proposed mechanistic framework linking the loss of PGM1 to Z-disk disruption via LDB3, and mitochondrial dysfunction, is derived primarily from multi-omics analyses and in vitro experiments. Additional experiments, including PGM1 overexpression or targeted perturbation of LDB3, will be required to directly test the relationships suggested by our data. Moreover, the current iPSC-derived model does not permit definitive determination of whether mitochondrial and sarcomeric dysfunction occur independently or whether mitochondrial impairment arises secondary to structural abnormalities. These limitations will be addressed in future studies.

In addition, while untargeted proteomics and electrophysiological analyses suggest sarcomere function is affected in PGM1-deficient iCMs, the absence of direct force or sarcomere contractility measurements represents an important limitation of the current 2D-based immature iPSC-CM model. Therefore, additional studies directly assessing sarcomere structure and function in a better-defined, mature cardiomyocyte model (such as an PGM1-deficient in vivo model), would be necessary to further confirm the deleterious effect of PGM1 deficiency on sarcomere function and structure. Furthermore, since our analyses were restricted to (immature) cardiomyocytes they do not capture potential contributions from other cardiac cell types or multicellular interactions present in vivo.

Finally, as is common in studies of ultra-rare diseases and iPSC-based models, sample size in our study was limited. To mitigate this, we employed multiple orthogonal approaches, including proteomics, metabolomics, functional assays, and in vitro modeling, and studied iCMs derived from four unrelated individuals carrying distinct pathogenic *PGM1* variants. The consistency of findings across cell lines and concordance with prior observations in PGM1-deficient mouse models and human cardiac tissue support the robustness and generalizability of our conclusions despite these constraints.

## Supplementary Information

Below is the link to the electronic supplementary material.

Supplementary Material 1

Supplementary Material 2

## Data Availability

All reagents are commercially available. Apart from the hiPSC lines, this study did not generate unique new reagents. Further information and requests for resources and/or reagents can be directed to the lead contact (tamas.kozicz@mssm.edu).

## Data availability

Deidentified metabolomics data have been deposited at National Metabolomics Data Repository (NMDR) Metabolomics workbench and are publicly available as of the date of publication. Deidentified mass spectrometry glycoproteomics and proteomics data have been deposited to the ProteomeXchange Consortium via the PRIDE partner repository. This study does not report a novel code. All the data reported in this paper such as values and complete statistical analysis used to create graphs in this paper are available as the additional data. All data and algorithms generated in this study can be requested from the corresponding author (Tamas Kozicz). Any additional information needed to analyze the data can be received from the lead contact upon request.
