# Supplementary material for: PGM1 deficiency is linked to sarcomeric and mitochondrial dysfunction in patient-derived iPSC-cardiomyocytes
Source: J Transl Med. 2026 Feb 21;24:430. doi: 10.1186/s12967-026-07808-9 (PMC13032676; doi:10.1186/s12967-026-07808-9)
Supplement: Supplementary file 1 — Supplementary Material 1 [file 12967_2026_7808_MOESM1_ESM.docx]

**PGM1 deficiency disrupts sarcomere and mitochondrial function in a stem-cell cardiomyocyte model**

Silvia Radenkovic, PhD^1,2*^, Graeme Preston, PhD^1,3*^, Rohit Budhraja, PhD^4^, Irena Muffels, MD, PhD^3^, Anna Ligezka, PhD^1^, Nathan P. Staff, MD, PhD^5^, Ron Hrstka^5^, Biijina Balakrishnan, PhD^6^, Rameen Shah, PhD^1,3^, Sanne Verberkmoes^1^, Ibrahim Shammas, MD^1^, Inez Bosnyak^1,8^, Kyle M. Stiers^9^, Kent Lai, PhD^6^, Lesa J. Beamer, PhD^,9^ Akhilesh Pandey, MD, PhD^4,7^, Eva Morava, MD, PhD^1,3,8^*, Tamas Kozicz, MD, PhD^1,3,10^*

^1^Department of Clinical Genomics, Mayo Clinic, Rochester, MN 55905, USA

^2^Department of Genetics, Section Metabolic Diagnostics, UMC Utrecht, Utrecht 3584 EA, NL

^3^Department of Genetics and Genomics Sciences, Icahn School of Medicine at Mount Sinai, New York City, NY 10029, USA

^4^Department of Laboratory Medicine and Pathology, Mayo Clinic, Rochester, MN 55905, USA

^5^Department of Neurology, Mayo Clinic, Rochester, MN 55905

^6^Department of Medical Genetics, University of Utah, Salt Lake City, UT 88413, USA

^7^Manipal Academy of Higher Education (MAHE), Manipal, Karnataka 576104, India

^8^Department of Biophysics, University of Pecs Medical School, 7624 Pecs, Hungary

^9^Biochemistry Department, University of Missouri, Columbia, MO 65211, USA

^10^Department of Anatomy, University of Pecs Medical School, 7624 Pecs, Hungary

*Authors which share the same-authorship position

Corresponding author: Tamas Kozicz, MD, PhD. [Tamas.kozicz@mssm.edu](mailto:Tamas.kozicz@mssm.edu)

**Supplementary (additional) material**

Additional material contains figures related to the work in the main text. Supplemenary workbook contains raw data generated from each omics analysis and statistical analysis.

**Supplementary methods**

**Immunohistochemistry**

German 2-well chamber slide systems (ThermoFisher Scientific, 154852) were coated with 1mg/ml Geltrex. iCMs were thawed as described above and approximately 2 million cells were plated in each well (day 0). The following day, cells were washed with DPBS and fresh RPMI+ medium added. The medium change was performed every two days. The beating was observed in all cells after 5 days in culture. On day 7 of the experiment, iCMs were washed with 3 times with TBS (Biorad, 1706435), fixed with 4% paraformaldehyde (Sigma, 162048) for 15 minutes at RT, and rinsed with 3xTBS. The cells were then incubated for 30 min RT in blocking solution containing 0.5 % Triton X-100 (Roche, 10789704001) plus 6% goat serum (Invitrogen, 31873) in TBS. Then, the blocking solution was removed and the cell incubated overnight at 4°C in primary antibodies diluted in the blocking solution (actinin mouse, ThermoFisher A7811, 1:200, cTnT (R) (Abcam ab45932) 1:200, alpha-connexin43 (Sigma C6219) 1:600). Incubate at 4 degrees C overnight. The following day, the cells were washed 3x TBS and then incubated with the secondary antibodies (1:400, anti-mouse IgG- AlexaFluor488, ThermoFisher A28175), anti-rabbit IgG-AlexaFluor 555, ThermoFisher, A31572) at 37°C in the dark for 1h. Next, the cells were washed 3X TBS, and the TBS aspirated completely before removing the chamber sides. Gold anti-fade reagent with DAPI was added to the glass bottom and topped with a glass coverslide before sealing. The cells were visualized by confocal microscope using appropriate lasers.

**Quantitative PCR analysis to assess cardiac-specific marker expression**

Briefly, 4 million iCMs were plated in RPMI+ insulin supplemented with 10% KOSR and 10µM ROCK inhibitor in 6-well plates. The following day, the cells were washed with DPBS and the medium refreshed. The medium was refreshed every 48h, for seven days. To isolate the RNA, the cells were dissociated from the plates with TrypLE (Thermo Fisher Scientific, A1217703), and collected in RPMI+insulin supplemented with 10% Knock-Out Serum (KOSR, ThermoFisher 10829018) and 10 µM ROCK inhibitor Y-27632. Then, the cells were centrifuged at 800 rpm, 6 min, RT. The supernatant was aspirated and the cells washed with 1mL DPBS, centrifuged again at 1500 rpm, 5 min, RT and the pellet snap frozen on dry ice and placed in -80°C freezer. For RNA isoltation from hiPSC, once hiPSC reached 80-90 % confluence in 60 mm plates, cells were washed with 3 ml DPBS, then 1ml of ReleSR was added. ReleSR was aspirated and the cells incubated for 5 min at 37 degrees. Next, the cells were harvested in 2mL mTeSR plus, centrifuged at 800 rpm, 6 min, RT. The supernatant was aspirated and the cell pellet washed with 1mL DPBS. The cells were centrifuged again at 1500 rpm, 5 min, RT, the supernatant was aspirated again and the cells snap frozen on dry ice and placed in -80°C freezer.

RNA mini plus isolation kit (Quiagen, 74134) was used to isolate RNA from the cell pellets and the RNA purity and concentration assessed by Nanodrop Spectrometer (ThermoFisher). Primer mix was prepared for the genes of interest (*cTNT, MYH7, MYL7, IDT*) and housekeeping gene (*ACTB, 18SRNA IDT*) by adding 1 μL of forward and reverse primer, 5 μL SYBR universal PCR master mix buffer (Applied biosciences), and 2 μL of RNase-free water to 1 μL of cDNA. The samples were transferred to a 324-well PCR plate, the plate was sealed and briefly centrifuged before running the assay on the Lightcycler RT-PCR system (Roche). Following protocol was used: 1) preincubation at 95°C for 5 min; 2) 45 amplification cycles at 95 °C, hold 10s; 60 °C, hold 10 s, 72 °C, hold 10 s; 3) melting curve 95 °C, hold 5s, 65°C, hold, 60 s, 97°C, continuous; 4) cooling at 40°C, hold 30 s. . The melt curve analysis was performed and the Ct values were exported from the program and analyzed. The 2ct method was used to analyze the relative changes in gene expression normalized against house-keeping gene mRNA expression^1^

**Multi-Electrode array (MEA)**

Multi-electrode array plates (MEA, Multichannel systems) were coated with fibronectin and incubated for 1h at 37°C. Then, 350.000 cells per well were plated in RPMI+ medium. The cells were left to incubate at RT for 1h, before transferring them to the incubator, to ensure optimal seeding. After 24h, the cells were observed and the medium was changed, after which the medium was changed every 48h and iCM observed for beating daily. Once the beating was observed, MEA plates were transferred to the multi-well MEA system (Multichannel systems), kept at 37°C. The signal was recorded for 2min using MEA multichannel-screen software (V 2.20.9, Multichannel systems). The data from multiple wells was analyzed using MEA multichannel analyzer software (V 2.0.6.0 Multichannel systems).

**iCardiomyocyte Contractility**

iCM contractility was assessed using the xCELLigence RTCA CardioECR System (Agilent). A 48-well CardioECR E-Plate was coated with 500 ug fibronectin from bovine plasma (Sigma). iCMs from an individual with PGM1 and a control were seeded 50,000 cells/well and cultured in RPMI-1640 media supplemented with B27 supplement, antibiotic-antimycotic, and 50 ug/mL uridine. After 7 days, well impedance was measured 1 ms-1 for 20 seconds.

**iCMs lysis and protein digestion**

PGM1-deficient and healthy control iCM were first solubilized in 8 M urea (in 100 mM TEAB buffer) supplemented with 1% protease inhibitor cocktail (Thermo Scientific) and then sonicated with a tip sonicator at 30% amplitude for 3 cycles of 10 seconds each. Cells were centrifuged at high speed to remove the cell debris. Protein amount was estimated by BCA assay as per the manufacturer’s instructions (Thermo Scientific). Equal amounts of protein from both groups were first reduced with 10 mM dithiothreitol (Sigma-Aldrich, USA) at 37º C followed by alkylated with 40 mM iodoacetamide (Sigma-Aldrich, USA) at room temperature in dark. The proteins were then digested with 1:20 w/w (protein: trypsin) ratio of trypsin (Worthington, USA) at 37º C overnight. Resulting peptides were cleaned up using C18 cartridges and labeled with tandem mass tags (TMT) (Thermo Fisher Scientific, USA) as per the manufacturer’s protocol.

**Peptide fractionation**

After checking the TMT labeling efficiency, the samples were subsequently pooled. Pooled peptides were subsequently split into two aliquots. One aliquot containing about 20% of total peptides was resuspended in solvent A (5 mM ammonium formate, pH 9) and fractionated by basic pH reversed phase liquid chromatography (bRPLC) on a C18 column (5 µm, 4.6 × 100 mm column, Waters) using a linear gradient of solvent B (5 mM ammonium formate, pH 9, in 90% acetonitrile) for 120 min on the Ultimate 3000 UHPLC system. Ninety-six fractions were collected and subsequently concatenated into 12 fractions. These concatenated 12 fractions were lyophilized and resuspended in 0.1% formic acid for liquid chromatography-tandem mass spectrometry (LC-MS/MS) for the proteomics study.

**Glycopeptide enrichment**

The other aliquot of total peptides containing the remaining 80% peptides was resuspended in 0.1% formic acid and injected into Superdex peptide 10/300 column (GE Healthcare) as described previously^2–4^. The peptides were separated using an isocratic flow of 0.1% formic acid for 130 min and 12 early fractions were collected starting at 10 minutes after injection for N- glycoproteomics study. The fractions were lyophilized and resuspended in 0.1% formic acid for LC-MS/MS analysis for the N-proteomics study.

**Liquid chromatography tandem mass spectrometry (LC-MS/MS)**

LC-MS/MS analysis of fractionated and enriched samples from both proteomics and N-glycoproteomics was carried out as previously described^2–4^ with some modifications. Peptides were then separated by liquid chromatography on an EASY-Spray column (75 m × 50 cm, PepMap RSCL C18, Thermo Fisher Scientific) at a flow rate of 300 nl/min for 150 min using a linear gradient of 0.1% formic acid in water (solvent A) and 0.1% formic acid in acetonitrile (solvent B). The samples were analyzed on an Orbitrap Exploris 480 mass spectrometer (Thermo Fisher Scientific) equipped with Ultimate 3000 liquid chromatography system (Thermo Fisher Scientific Inc.). All experiments were done in DDA mode at an isolation window of 0.7 m/z. Precursor ions were acquired in the Orbitrap mass analyzer in m/z range of 300-1,700 for proteomics and 350-2,000 for N-glycoproteomics. Precursor ions were acquired at a resolution of 120,000 (at m/z 200) and fragment ions at a resolution of 30,000 (at m/z 200). Precursor fragmentation was carried out using normalized higher-energy collisional dissociation (HCD) method of 34 for proteomics and normalized stepped HCD at 15, 25 and 40% for glycoproteomics.

**Targeted tracer metabolomics experiments**

Briefly, approximately 1million iCMs per well was plated on Geltrex coated 24-well plates in triplicates (day 0). The following day, the medium was refreshed (day 1). The medium was refreshed again after 48h (day 3). By day 5, all iCardiomyocyte cell lines were beating. The cells were washed with DPBS, and incubated with RPMI+insulin medium (ThermoFisher, 221704) supplemented with A) 5.5mM (physiologic concentration) ^13^C_6_-glucose, or B) 5.5mM ^12^C_6_-glucose. The cells were incubated for additional 48 h to ensure steady state labeling of the cells has occurred and metabolite extraction was performed as described below.

Briefly, cells were washed with ice-cold saline solution, 200 microL ice-cold extraction buffer (80 % MeOH, IS) was added each well. After 2 min, the cells were scraped in extraction buffer, transferred to fresh Eppendorf tubes and placed overnight at -80 °C. Next, all the samples were pelleted at 15,000 rpm for 20min at 4 °C. Supernatant containing metabolites was transferred to a new Eppendorf tube and metabolites measured with LC/MS (see below). 100 µL of 200mM NaOh was added to the cell pellets. The pellets were then incubated at 95 °C for 30min, centrifuged at 5000 rpm, rpm for 10min at 4 °C. Finally, the supernatant containing proteins was used for protein concentration determination with BCA Pierce Protein Assay kit (ThermoFisher, A55865).

Previously described method was used to analyze metabolites by LC/MS ^4–7^. Briefly, 10 µL of sample was separated on a C18 ion-pairing liquid chromatography column and the metabolites resolved by Thermo Fisher Q-Exactive Hybrid Quadrupole Orbitrap MS in negative ion mode (resolution 140,000 at 200 m/z, AGC at 3e^6^, 512 ms ion fill time, full scan 70-850 m/z). The ESI settings were set to: 50 sheet gas flow rate, auxiliary gas flow rate 15, spray voltage of 4 kV, S-lens RF level of 60, and the capillary temperature at 350°C. Metabolite identification was performed according to their elution times, m/z ratio and in-house metabolite standard library. Peak picking and correction of naturally occurring carbon isotops was performed by El-Maven v0.12.0/Polly ^TM^Labeled LC-MS Workflow^8^. Metabolite abundances were normalized to protein concentration and internal standards. Absolute quantification was not performed. Relative values were established using healthy control organoids as reference.

**Additional Figures**


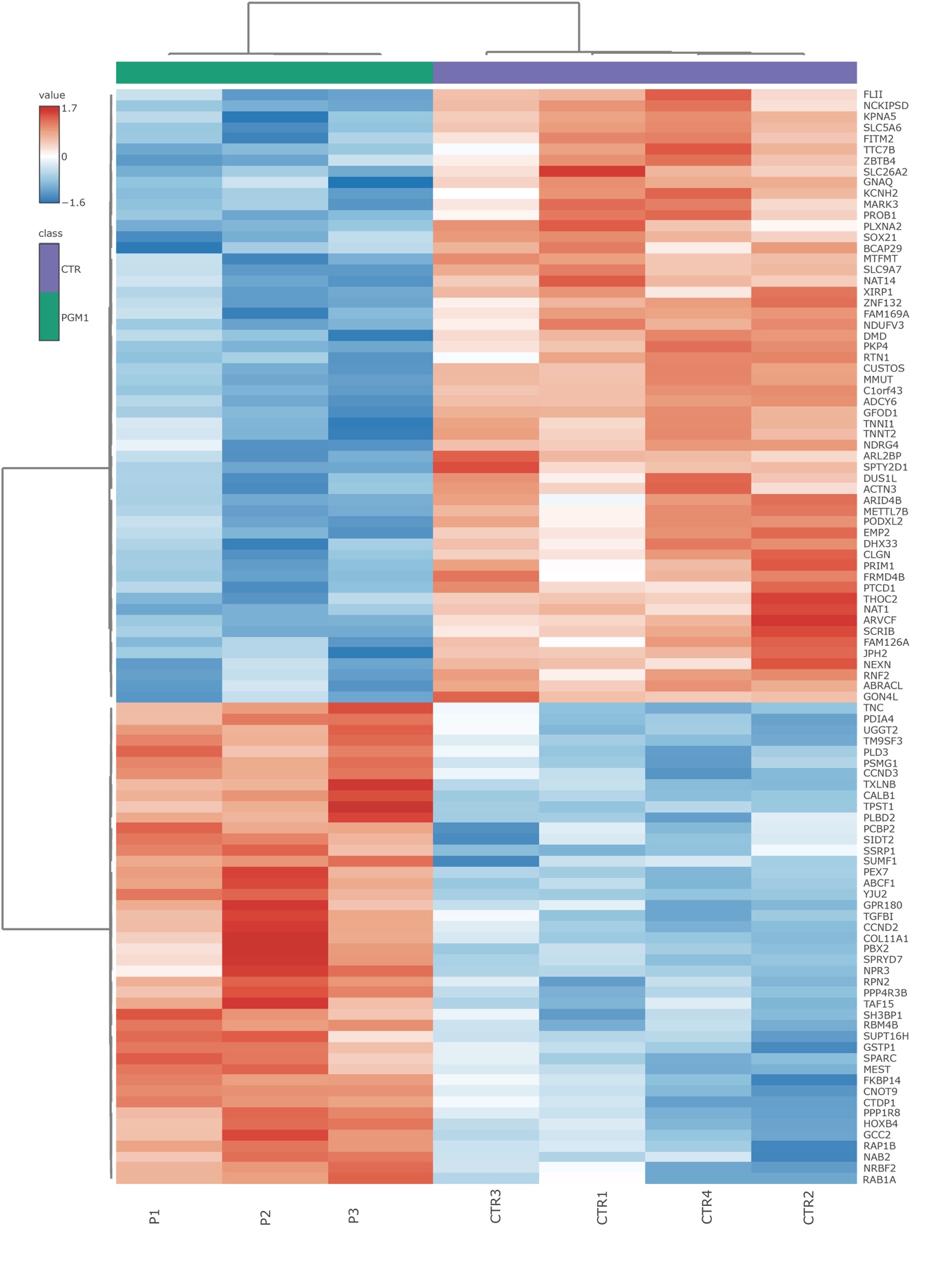


**Additional Figure 1.** Heatmap of top 100 significantly (p-value < 0.05) upregulated and downregulated proteins in PGM1-deficient iCM PGM1 n=3, t=1; CTR n=4, t=1.

**
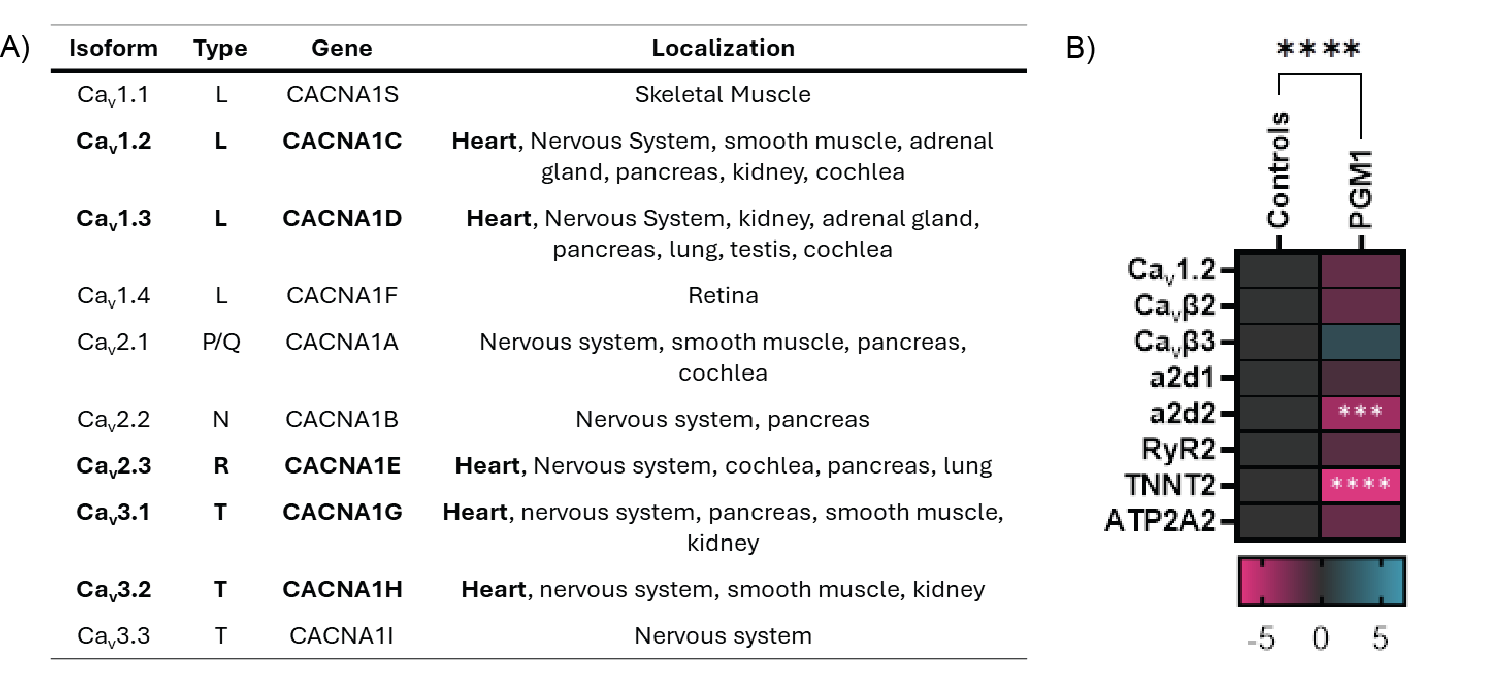
**

**Additional Figure 2.** Some of the cardiac excitation-contraction coupling related proteins and calcium homeostasis are affected in PGM1-deficient iCMs. A) The list of the proteins involved in cardiac excitation-contraction and calcium homeostasis. B) the relative expression and statistical significance of identified proteins in PGM1-deficient vs. CTR cardiomyocytes.


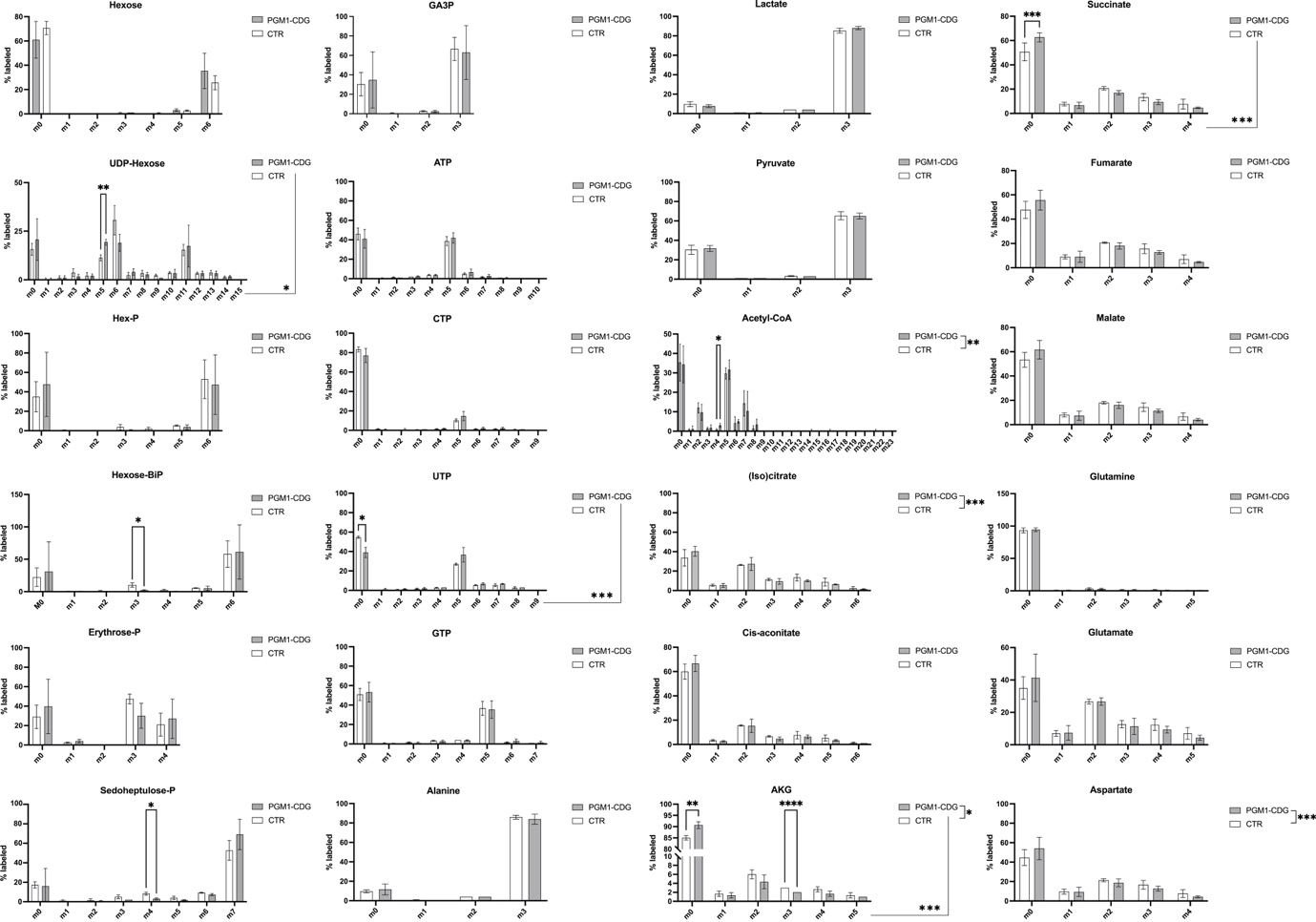


**Additional Figure 3.** Isotopologue distribution (positional labeling) of ^13^C_6_-glucose in specific metabolites belonging to glucose and galactose metabolism, pentose phosphate pathway, nucleotide-phosphates, TCA cycle and glutamine metabolism. m(0-n) represents the number of carbons labeled by ^13^C_6_-glucose, where n=number of carbons present in the metabolite. Two-way ANOVA and multiple comparisons with Šidak correction was performed. Significant p-value is indicated in * (* p<0.05; ** p< 0.01; *** p<0.001). (PGM1 n=3; t=2-3; CTR n=3, t=2-3). Detailed statistical analysis is provided in Additional data.


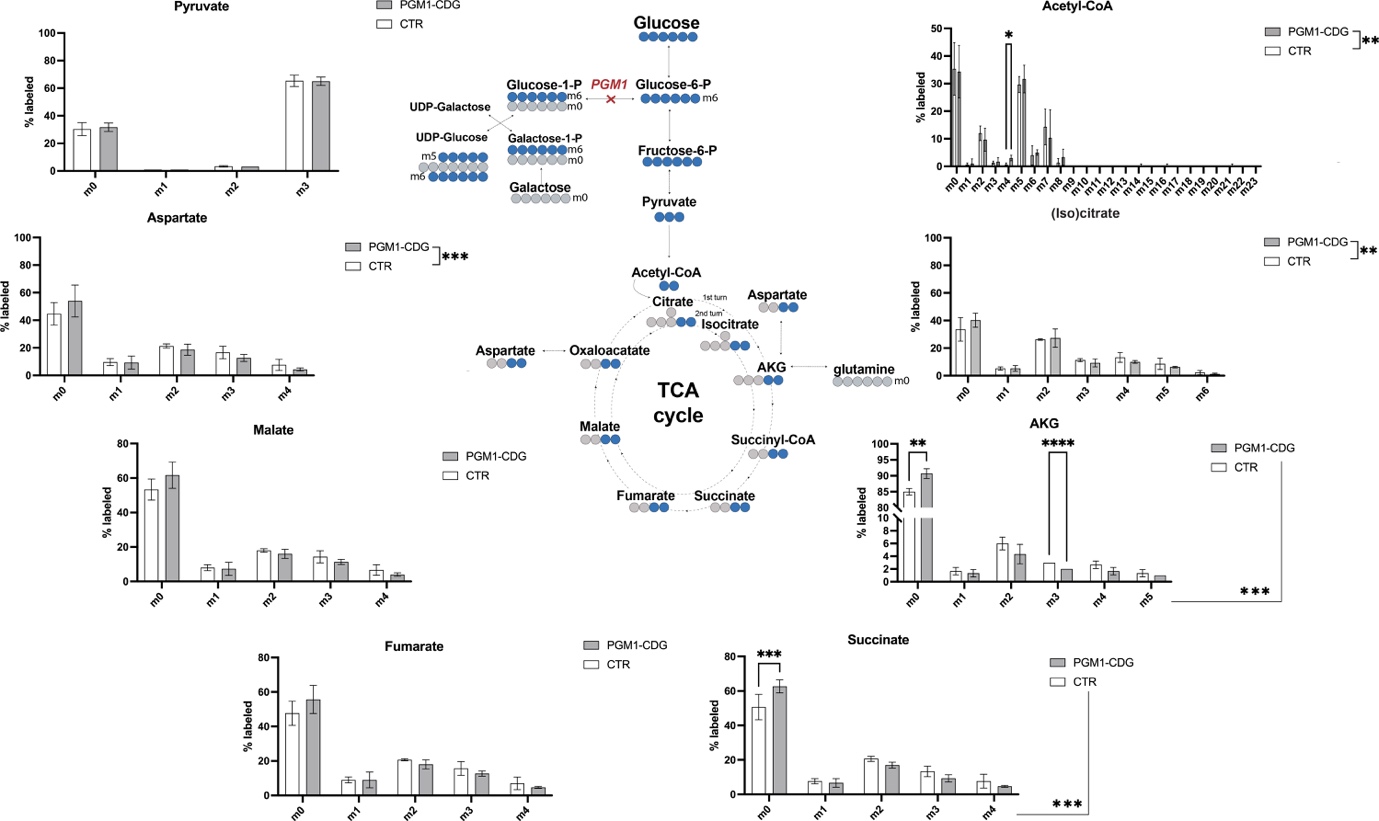


**Additional Figure 4.** Isotopologue distribution (positional labeling) of ^13^C_6_-glucose in related to the TCA cycle. m(0-n) represents the number of carbons labeled by ^13^C_6_-glucose, where n=number of carbons present in the metabolite. Two-way ANOVA and multiple comparisons with Šidak correction was performed. Significant p-value is indicated in * (* p<0.05; ** p< 0.01; *** p<0.001). (PGM1 n=3; t=2-3; CTR n=3, t=2-3). Detailed statistical analysis is provided in Additional data.


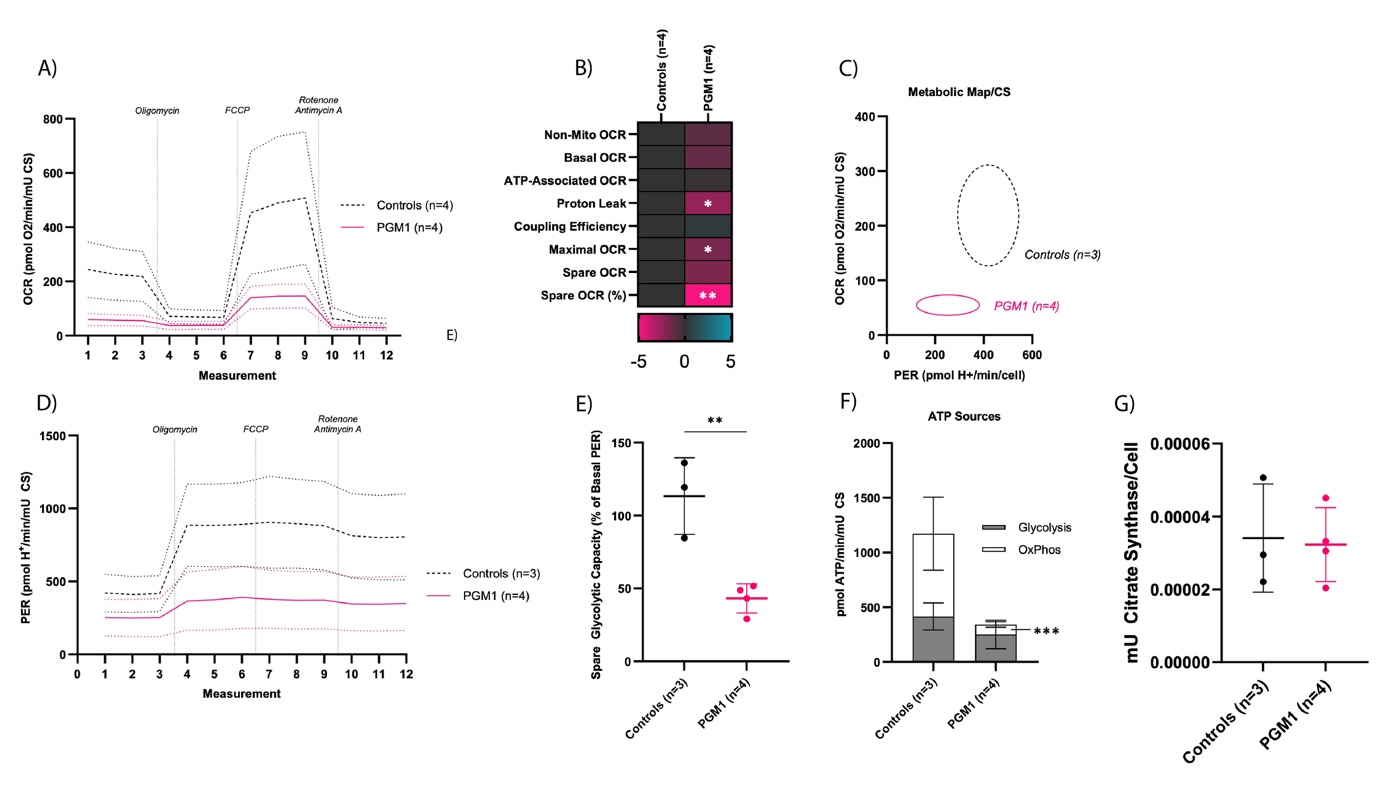


**Additional Figure 5.** Mitochondrial stress test normalized to Citrate Synthase (CS). A) Oxymetry plot displaying Oxygen Consumption Rate (OCR) of PGM1 and Control iCMs (mean +/- SD) during Mito Stress Test Assay. B) Heat map of Mito Stress Test OCR readouts, representing SDs from the mean of the controls. C) Proton efflux rate (PER) plot displaying PER of PGM1 and Control iCMs (mean +/- SD) during Mito Stress Test Assay. D) Metabolic map displaying Proton Efflux Rate (PER) on the X axis and OCR on the Y axis. Circles represent SD of the means of each group. E) Spare glycolytic capacity. F) Bar plot displaying sources of ATP. G) Citrate Synthase (CS) activity normalized to cell count. The P values are indicated as significant * (<0.05), ** (<0.01), or *** (<0.001). (PGM1 n=4, t=3 CTR n=3, t=3).

**Supplementary references**

**1**. Pfaffl, M. W. *A New Mathematical Model for Relative Quantification in Real-Time RT-PCR*. *Nucleic Acids Research* vol. 29 (2001).

2. Budhraja, R. *et al.* N-glycoproteomics reveals distinct glycosylation alterations in NGLY1-deficient patient-derived dermal fibroblasts. *J Inherit Metab Dis* 46, 76–91 (2023).

3. Balakrishnan, B. *et al.* AAV-based gene therapy prevents and halts the progression of dilated cardiomyopathy in a mouse model of phosphoglucomutase I deficiency (PGM1-CDG). *Translational Research* https://doi.org/10.1016/j.trsl.2023.01.004 (2023) doi:10.1016/j.trsl.2023.01.004.

4. Radenkovic, S. *et al.* Neural and metabolic dysregulation in PMM2-deficient human in vitro neural models. *Cell Rep* 43, (2024).

5. Radenkovic, S. *et al.* TRAPPC9-CDG: A novel congenital disorder of glycosylation with dysmorphic features and intellectual disability. *Genetics in Medicine* https://doi.org/10.1016/j.gim.2021.12.012 (2022) doi:10.1016/j.gim.2021.12.012.

6. Radenkovic, S. *et al.* Tracer metabolomics reveals the role of aldose reductase in glycosylation. *Cell Rep Med* 4, (2023).

7. Radenkovic, S. *et al.* The Metabolic Map into the Pathomechanism and Treatment of PGM1-CDG. *Am J Hum Genet* 104, 835–846 (2019).

8. Agrawal, S. *et al.* El-MAVEN: A Fast, Robust, and User-Friendly Mass Spectrometry Data Processing Engine for Metabolomics. in 301–321 (2019). doi:10.1007/978-1-4939-9236-2_19.
